# The microdissected gene expression landscape of nasopharyngeal cancer reveals vulnerabilities in FGF and noncanonical NF-κB signaling

**DOI:** 10.1126/sciadv.abh2445

**Published:** 2022-04-08

**Authors:** Joshua K. Tay, Chunfang Zhu, June Ho Shin, Shirley X. Zhu, Sushama Varma, Joseph W. Foley, Sujay Vennam, Yim Ling Yip, Chuan Keng Goh, De Yun Wang, Kwok Seng Loh, Sai Wah Tsao, Quynh-Thu Le, John B. Sunwoo, Robert B. West

**Affiliations:** 1 Department of Pathology, Stanford University School of Medicine, Stanford, CA, USA.; 2 Department of Otolaryngology–Head and Neck Surgery, Stanford University School of Medicine, Stanford, CA, USA.; 3 Department of Otolaryngology–Head & Neck Surgery, National University of Singapore, Singapore, Singapore.; 4 School of Biomedical Sciences, Li Ka Shing Faculty of Medicine, The University of Hong Kong, Hong Kong, China.; 5 Department of Radiation Oncology, Stanford University School of Medicine, Stanford, CA, USA.

## Abstract

Nasopharyngeal cancer (NPC) is an Epstein-Barr virus (EBV)–positive epithelial malignancy with an extensive inflammatory infiltrate. Traditional RNA-sequencing techniques uncovered only microenvironment signatures, while the gene expression of the tumor epithelial compartment has remained a mystery. Here, we use Smart-3SEQ to prepare transcriptome-wide gene expression profiles from microdissected NPC tumors, dysplasia, and normal controls. We describe changes in biological pathways across the normal to tumor spectrum and show that fibroblast growth factor (FGF) ligands are overexpressed in NPC tumors, while negative regulators of FGF signaling, including SPRY1, SPRY2, and LGALS3, are down-regulated early in carcinogenesis. Within the NF-κB signaling pathway, the critical noncanonical transcription factors, RELB and NFKB2, are enriched in the majority of NPC tumors. We confirm the responsiveness of EBV-positive NPC cell lines to targeted inhibition of these pathways, reflecting the heterogeneity in NPC patient tumors. Our data comprehensively describe the gene expression landscape of NPC and unravel the mysteries of receptor tyrosine kinase and NF-κB pathways in NPC.

## Teaser

Microdissected gene expression reveals susceptibility of nasopharyngeal cancer to inhibition of noncanonical NF-kB signaling.

## INTRODUCTION

Nasopharyngeal cancer (NPC) is an Epstein-Barr virus (EBV)–positive epithelial malignancy endemic to several populations around the world, including the Southern Chinese, Southeast Asians, North Africans, and the Alaskan natives (*1*–*3*). The establishment of type II EBV latency is an early event in carcinogenesis (*4*–*7*) and is accompanied by elevated EBV immunoglobulin A (IgA) serology titers (*5*, *8*), reflecting an EBV-associated inflammatory response in NPC tumors.

The inflammatory microenvironment infiltrate accounts for as much as 70 to 80% of the NPC tumor volume, resulting in challenges in identifying molecular signals distinct to the tumor epithelial compartment (*9*, *10*). Furthermore, the amount of available biological material is limited, as the treatment for primary tumors is with chemoradiotherapy, rather than surgery. Nasopharyngeal diagnostic biopsies are performed using a transnasal endoscopic approach and measure only 3 to 4 mm in size.

Whole-exome sequencing studies have thus relied on stringent pathological selection and an increased depth of sequencing to identify mutational changes in NPC (*11*–*13*). It was first observed that NPC tumors have a lower mutational burden compared with other cancers, with mutations scattered among several pathways including chromatin modification and receptor tyrosine kinase (RTK) signaling (*11*). These mutations were sparsely distributed among downstream mediators of the phosphatidylinositol-3-kinase/protein kinase B (PIK3CA/AKT) and mitogen-activated protein kinase (MAPK) pathways, as well as occasional mutations in Erb-B2 receptor tyrosine kinase 2/3 (ERBB2/3) and fibroblast growth factor receptor 2 (FGFR2). Subsequently, a further group of mutations in negative regulators of the nuclear factor κB (NF-κB) signaling was observed in 7 to 41% of cases, suggesting that NF-κB activation is selected for during NPC pathogenesis (*12*, *13*). Despite these efforts, molecular targets that are clinically actionable in a majority of NPC tumors remain elusive.

Large gene expression studies of histologically pure NPC tumors have not been performed because of these challenges with tissue purity and quantity. Traditional approaches using RNA-sequencing (RNA-seq) of bulk NPC tumors identified tumor subtypes associated with proliferative or stromal tumor-infiltrating lymphocyte (TIL) signatures, which may be related to tumor cellularity (*14*). Tumors with a high TIL signature had an improved prognosis. Single-cell gene expression studies of NPC tumors have also identified substantial intertumor microenvironment heterogeneity, with high macrophage, dendritic cell, and natural killer cell signatures associated with improved survival outcomes (*15*, *16*). However, there has been limited insight into changes within the tumor epithelial compartment of NPC tumors. Here, we overcome these limitations by performing compartment-specific gene expression profiling of nasopharyngeal biopsies with laser-capture microdissection (LCM) and Smart-3SEQ (*17*), a 3′ RNA-seq technique for preparing transcriptome-wide gene expression profiles from just hundreds of microdissected archival cells in FFPE (formalin-fixed paraffin-embedded) tissue. To establish ground truth gene expression profiles of normal nasopharyngeal epithelium, we include panendoscopy biopsies (*18*, *19*) of normal nasopharyngeal epithelium and compare them with normal epithelium from other sites of the upper airway. Critically, to evaluate NPC tumor biopsies, we harvest cells from the tumor epithelia and microenvironment in separate microdissections, obtaining compartment-specific gene expression libraries. All slides were first reviewed by a board-certified pathologist to mark out the best areas for microdissection targeting histologically pure areas. For tumors with a Regaud pattern comprising well-defined epithelial cells surrounded by lymphocytes and connective tissue, microdissection was performed following the distinct borders between the tumor compartments. In the case of challenging tumors with a Schmincke pattern comprising epithelial cells intermingled with the microenvironment, microdissections targeting smaller specific areas were performed in the presence of a pathologist for optimal accuracy. To increase the success of obtaining at least one high-quality gene expression library for tumor regions from each patient sample, biological replicates were prepared, targeting a separate area of tumor epithelium. We also sampled regions of dysplasia and histologically normal epithelium adjacent to the tumor, which allowed us to profile changes in gene expression across the spectrum of normal to tumor nasopharyngeal epithelium.

## RESULTS

**Fig. 1.**
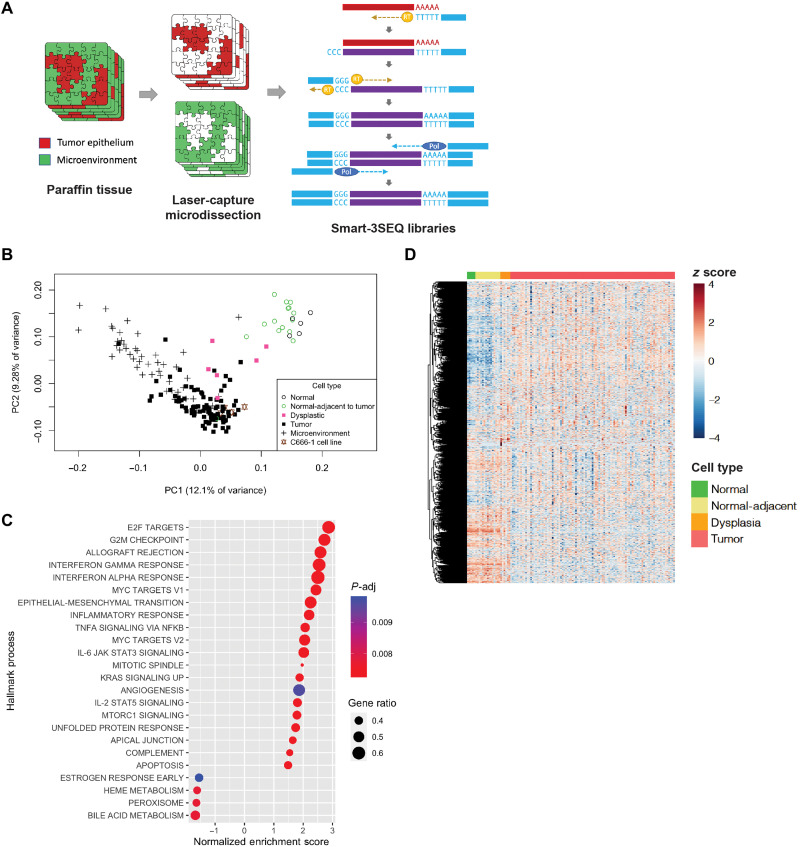
Hallmark biological processes are significantly altered in NPC. (**A**) LCM of tumor epithelial cells and the microenvironment separately, followed by gene expression libraries prepared by Smart-3SEQ. (**B**) Principal components analysis of human nasopharyngeal tissue gene expression from tumor and normal biopsies (*n* = 175 libraries). (**C**) Significantly altered Hallmark biological processes comparing tumor epithelial cells (*n* = 54) and normal nasopharyngeal epithelium (*n* = 5). (**D**) Heatmap of 2570 genes from Hallmark biological processes found to be significantly altered (*P*-adj < 0.05) across the normal tumor spectrum (*n* = 120 libraries).

We prepared, sequenced, and analyzed libraries from a total of 171 biologically unique regions from 67 nasopharyngeal biopsies (Fig. 1A, table S1, and fig. S1, A and B). Principal components analysis showed that normal nasopharyngeal epithelium from panendoscopy biopsies clustered together with histologically normal tumor-adjacent epithelium, while NPC tumor regions clustered separately, with dysplastic samples in between (Fig. 1B). We did not observe any significant batch effect within the top principal components, based on the batch of library preparation or the age of the tissue sample (fig. S2). When biological replicates of tumor epithelial regions were successfully profiled, principal components analysis and unsupervised hierarchical showed consistency between libraries prepared, with biological duplicates from the same patient clustering together (fig. S2, C and D).

To provide a global summary of the gene expression changes, we performed gene set enrichment analysis (GSEA) using Hallmark gene sets representing well-defined biological states (*20*). Consistent with malignancy, Hallmark processes involved in proliferation (E2F targets, G2M checkpoint, and MYC targets), angiogenesis, and epithelial-mesenchymal transition were among the most highly enriched in the tumor epithelial compartment compared with the normal nasopharynx (Fig. 1C and fig. S3). Hallmark pathways involved in immune response were also very highly enriched in tumor epithelium compared with normal, including interferon-α and interferon-γ response, interleukin-6 (IL-6)/Janus kinase (JAK)/signal transducer and activator of transcription 3 (STAT3) signaling, and NF-κB signaling (Fig. 1C). The enrichment of immune processes was an early event observed in normal-adjacent epithelium, with further enrichment in dysplasia, before diminishing in tumor (compared to dysplasia), suggesting a dampening of the inflammatory response in the late moments of carcinogenesis (fig. S3). Specific Gene Ontology (GO) biological processes that were highly enriched in tumors included viral response, immune response, and leukocyte migration, with the chemokines CXCL9, CXCL10, and CXCL11 among the most highly up-regulated (fig. S4, A and B). Unsupervised hierarchical clustering performed using genes from significantly altered Hallmark processes across the normal tumor spectrum showed distinct gene expression profiles based on cellular phenotypes (Fig. 1D). One cluster showed genes that were up-regulated in tumor cells but down-regulated in normal cells, while the other cluster showed the opposite. Dysplastic cells appeared to express genes in both cluster, supporting an intermediate transition state. A full list of differentially expressed genes and pathways comparing between cell types across the normal tumor spectrum, as well as the microenvironment, is included in the Supplementary Materials accompanying the manuscript.

### The nasopharyngeal epithelium is unique in the upper airway

It is interesting that latent EBV epithelial infection and the associated inflammatory characteristics are present in the majority of nasopharyngeal tumors but rarely observed in epithelial tumors from other parts of the upper airway. To address the predisposition of nasopharyngeal epithelium to this phenomenon, we compared the gene expression profile of the normal nasopharynx with an additional five epithelial sites of the upper airway obtained from normal panendoscopy biopsies. Principal components analysis showed distinct clustering of normal nasopharyngeal epithelium from the squamous epithelium present in other upper airway sites, with the first principal component accounting for 44.4% of variance between both groups (fig. S5A). Ciliary processes were highly enriched in nasopharyngeal epithelium, while processes involved in epithelial differentiation, including type II keratins (KRT4, KRT5, KRT6A, and KRT6B), were disenriched compared with squamous epithelial sites (fig. S5, B and C). Notably, genes involved in the lymphocyte chemotaxis pathway were enriched in normal nasopharyngeal epithelium (*P-*adj = 0.0376; fig. S5, C to E), including the chemoattractant CCL20, a key recruiter of dendritic cells and macrophages into mucosal tissues through its action on the CCL20/CCR6 axis (*21*–*23*). Consistent with these observations, gene signature–based deconvolution of cell types by xCell (*24*) showed highly significant enrichment of immature dendritic cell (iDC) signatures in the normal nasopharynx compared with epithelium from other upper airway sites including the tonsil (*P* = 1.11 × 10^−10^; fig. S5F), consistent with the role of the nasopharynx as an important site for antigen presentation as part of Waldeyer’s ring. Early immunohistochemical studies have shown the presence of antigen-presenting cells within the epithelial crypts of normal nasopharyngeal epithelium (*25**)*.

### Fibroblast growth factor (FGF) signaling is activated in NPC

**Fig. 2.**
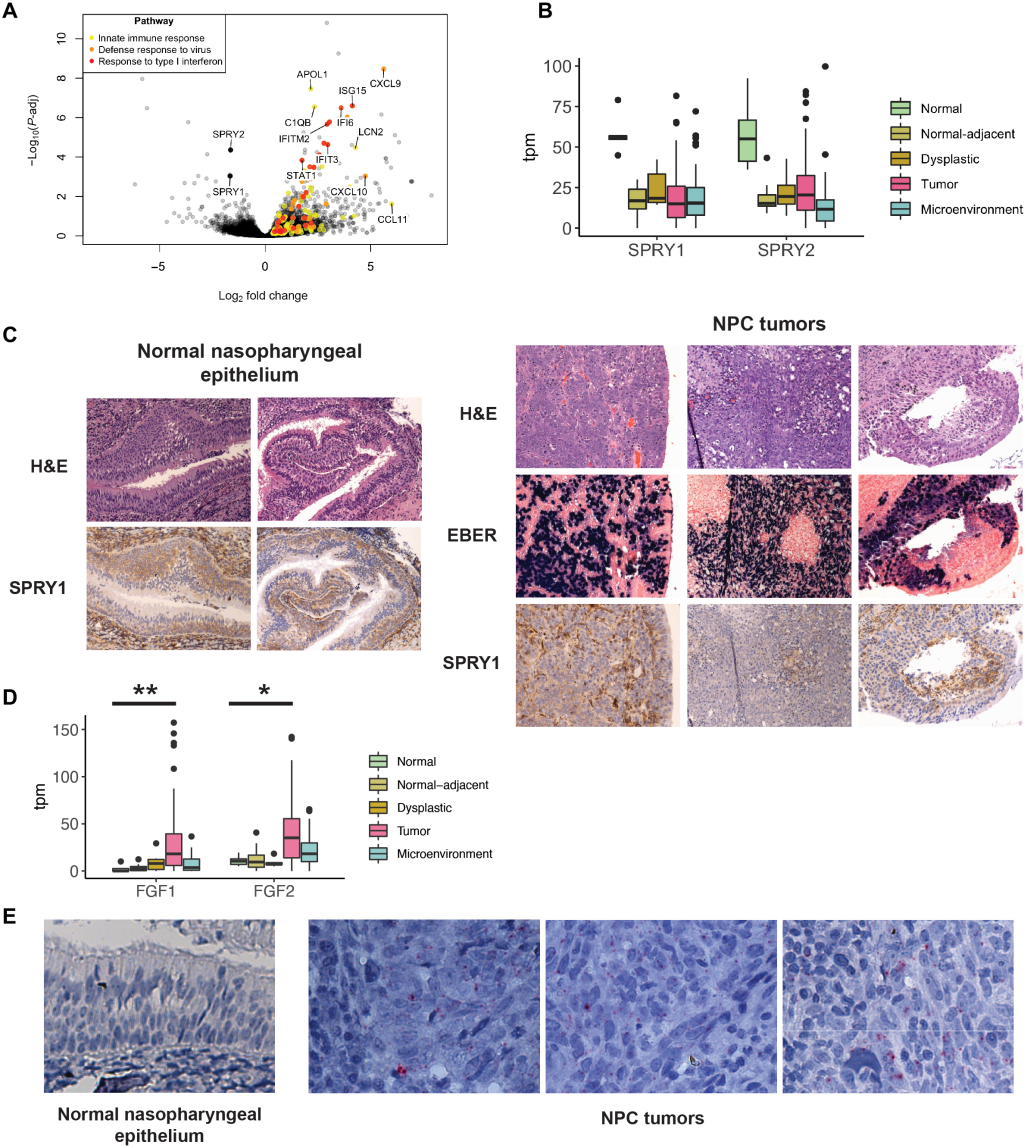
FGF signaling is enriched in NPC. (**A**) Volcano plot of early gene expression changes in NPC showing differentially expressed genes between normal-adjacent epithelium (*n* = 14 tumors) and normal nasopharyngeal epithelium samples (*n* = 5 controls). (**B**) Gene expression of SPRY1 and SPRY2 across the normal tumor spectrum (*n* = 171 libraries). (**C**) Immunohistochemistry of SPRY1 in normal nasopharyngeal epithelium and NPC tumor samples. (**D**) Expression of FGF1 and FGF2 across the normal tumor spectrum (*n* = 171 libraries) **P-adj = 0.00326; *P-adj = 0.0158. (**E**) FGF2 RNA ISH in normal nasopharyngeal epithelium and NPC tumor specimens.

To perform a balanced differentially expressed gene analysis in instances where biological duplicate libraries of the tumor epithelial compartment were obtained from the same patient, we selected only the library with the highest percentage mappability (table S1). While pathways associated with immune response were enriched in normal-adjacent epithelium, we observed that Sprouty proteins, important intracellular negative regulators of RTK signaling, were disenriched early in carcinogenesis. SPRY1 and SPRY2 were among the most highly down-regulated genes in normal-adjacent regions when compared with normal controls and remained down-regulated in dysplasia and tumor (Fig. 2, A and B). On immunohistochemistry, SPRY1 was highly expressed in the basal layer of normal nasopharyngeal epithelium. In contrast, the reduced protein expression of SPRY1 in NPC tumors corresponded to the EBV-positive cells in the tumor epithelial compartment (Fig. 2C).

Sprouty proteins inhibit signaling by binding and sequestering growth factor receptor bound protein 2 (GRB2), an adaptor protein linking growth factor receptors with downstream MAPK and PIK3CA/AKT pathways (*26*, *27*). We therefore considered the gene expression of RTK ligands and receptors, as these could be important upstream drivers of proliferation in NPC. Differential gene expression of growth factor ligands remarkably showed that FGF1 and FGF2 were among the most significantly up-regulated RTK ligands within the tumor epithelial compartment (Fig. 2D and fig. S7). In contrast, ligands of the epidermal growth factor (EGF) pathway were not up-regulated, apart from insulin-like growth factor 1 (IGF1). In this cohort of NPC tumors, 66.7 and 70.7% of tumors demonstrated increased FGF1 [transcripts per million (TPM) > 10.5] and FGF2 (tpm > 19.5) gene expression, respectively, when considering the highest threshold observed in normal samples. We confirmed via RNA in situ hybridization (ISH) that FGF2 RNA was present in tumor epithelial cells, absent in the microenvironment, and absent in normal nasopharyngeal epithelium (Fig. 2E).

Galectins are extracellular carbohydrate-binding proteins that regulate the distribution and function of membrane glycoproteins, including growth factor receptors. Galectin-1 has been shown to mimic FGF ligand and activate FGFR1, while Galectin-3 inhibits FGFR1 by inducing the overclustering of FGFR1 on the membrane surface and limiting downstream signaling (*28*). We observed up-regulation of Galectin-1 and down-regulation of Galectin-3 gene expression, which is consistent with activated FGF/FGFR signaling (fig. S6). Together, these changes in FGF pathway mediators suggest that NPC tumors might signal via FGF in an autocrine manner. We did not observe any significant up-regulation of other RTK ligands and receptors known to be inhibited by Sprouty proteins, including EGF, IGF, PDGF (platelet-derived growth factor), VEGF (vascular endothelial growth factor), and their receptors (fig. S7).

**Fig. 3.**
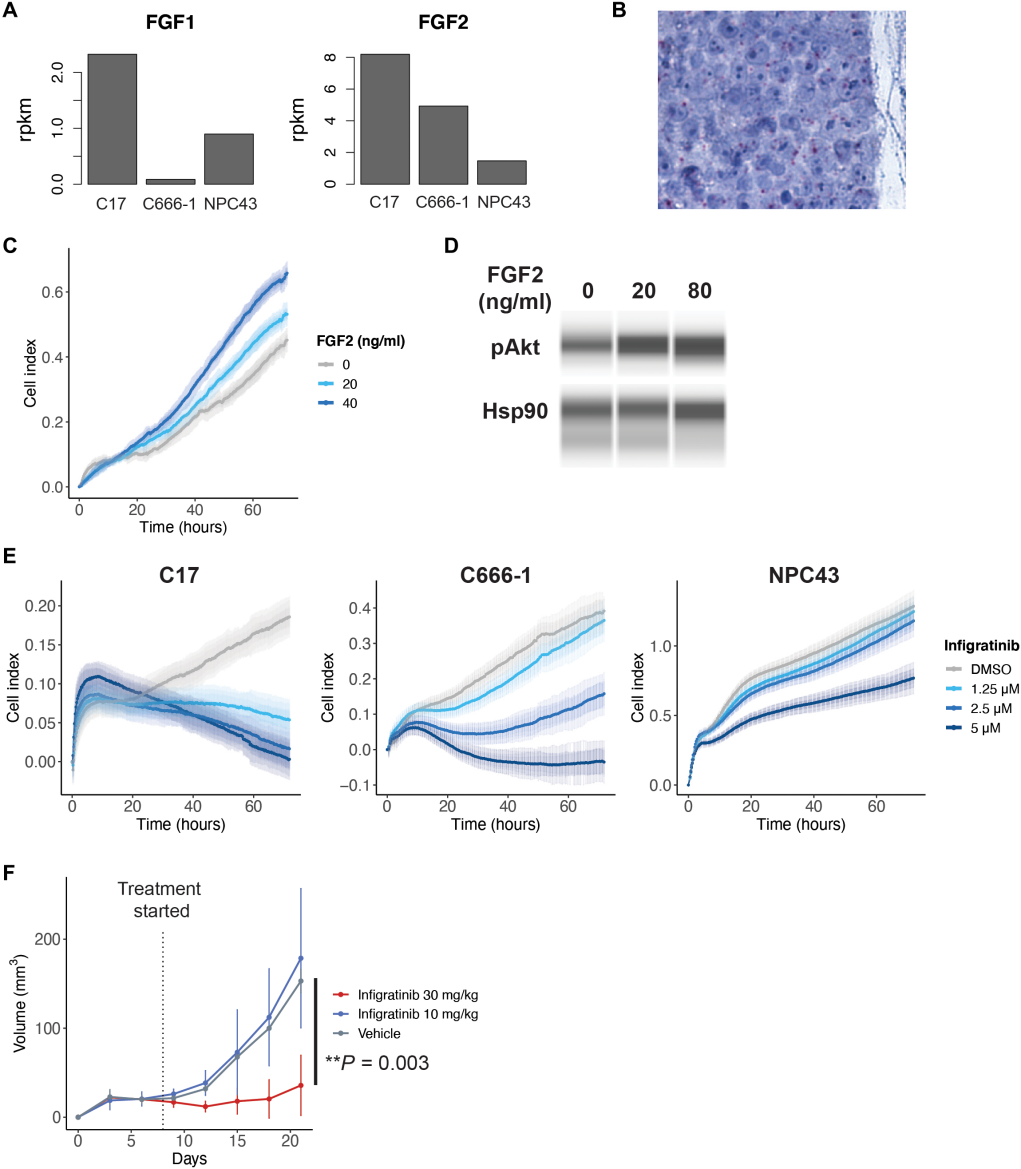
In vitro and in vivo inhibition of FGF signaling in NPC. (**A**) Gene expression of FGF1 and FGF2 in EBV-positive NPC cell lines (*n* = 1 per cell line). (**B**) RNA ISH of FGF2 in the C666-1 NPC cell line grown in organoid. (**C**) Proliferation of the C666-1 cell line in media supplemented with FGF2. (**D**) Western blot of AKT expression in C666-1 cells after treatment with FGF2. (**E**) In vitro proliferation of EBV-positive NPC cell lines when treated with infigratinib, an FGFR inhibitor, as measured on the xCELLigence real-time cell analyzer. Error bars represent SD. (**F**) Growth of C666-1 xenografts in NSG mice when treated with infigratinib.

We next modeled the relevance of FGF signaling in a panel of EBV-positive NPC cell lines: C17, C666-1, and NPC43, none of which have been observed to harbor FGFR mutations (*29*, *30*). Analysis of previous RNA-seq of these cell lines showed expression of FGF ligands, especially FGF2 in the C17 and C666-1 cell lines, which we confirmed on RNA ISH (Fig. 3, A and B). Addition of FGF2 to culture media resulted in increased growth of C666-1 cells and increased AKT phosphorylation (Fig. 3, C and D). Similarly, treatment with infigratinib, an FGFR1/2/3-specific inhibitor, resulted in reduced cell proliferation and increased cell death in a dose-response relationship. The inhibitory effect of infigratinib was greatest for the C17 cell line, followed by the C666-1 cell line, correlating with their level of FGF2 expression (Fig. 3E). We further confirmed the effect of FGFR inhibition in vivo, where treatment of C666-1 xenografts with infigratinib at 30 mg/kg showed reduced tumor growth (Fig. 3F).

### Tumor microenvironment relationships in NPC

**Fig. 4.**
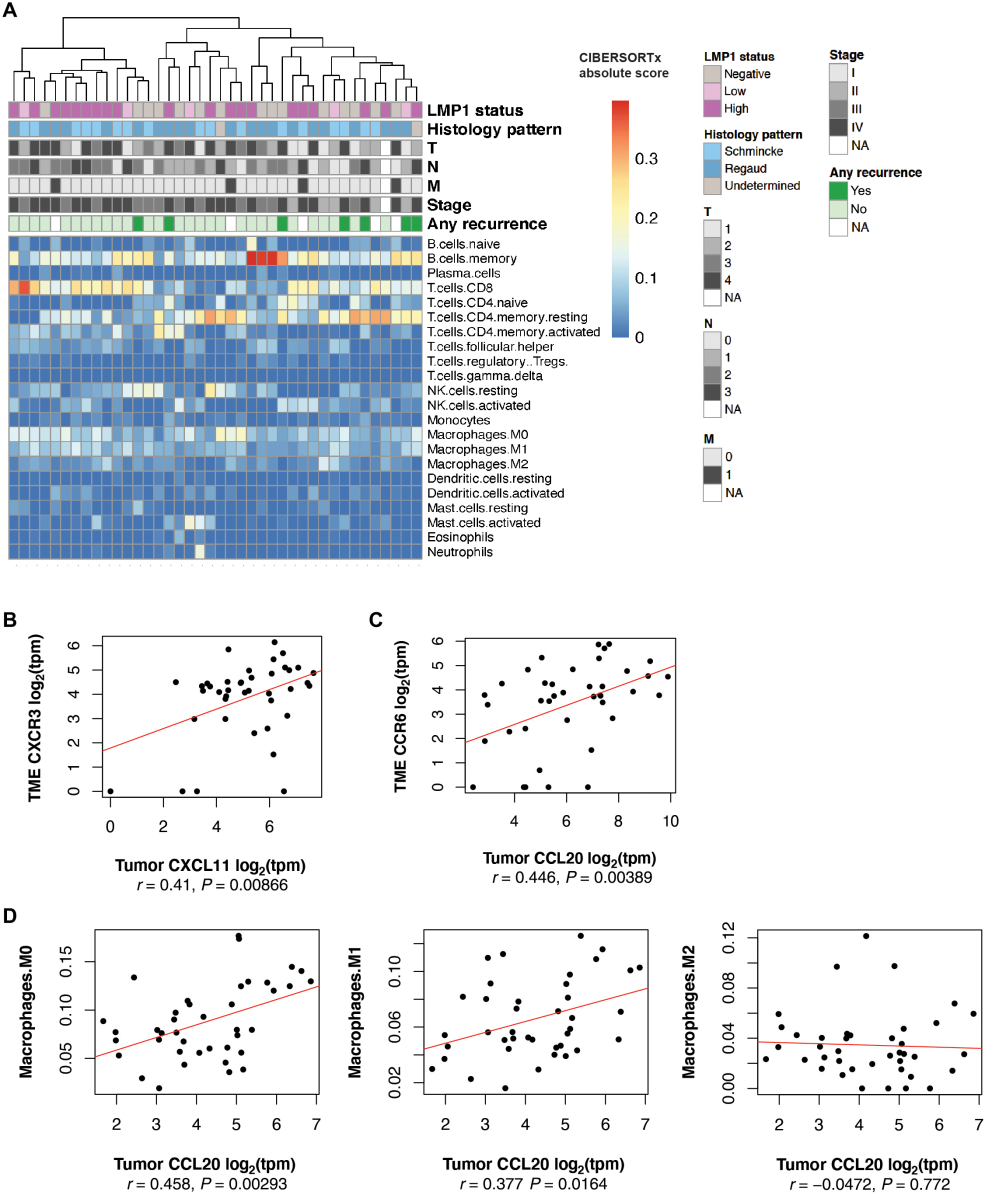
Tumor microenvironment relationships in NPC. (**A**) Heatmap of CIBERSORTx deconvoluted immune cell fractions in the microenvironment (*n* = 40 paired tumor epithelial and microenvironment libraries). (**B**) Correlation analysis of tumor CXCL11 expression and its receptor CXCR3 in the microenvironment (*n* = 41 paired libraries). (**C**) Correlation analysis of tumor CCL20 expression and its receptor CCR6 in the microenvironment (*n* = 41 paired libraries). (**D**) Correlation analysis of tumor epithelial CCL20 and CIBERSORTx deconvoluted M0, M1, and M2 macrophage fractions in the microenvironment (*n* = 41 paired libraries).

To better understand the cellular compositions within the inflamed tumor microenvironment, we applied CIBERSORTx, an in silico deconvolution approach to estimate the fractional compositions of 22 immune cells types, based on purified gene expression signatures (*31*). We observed that CD8 cytotoxic T cells, CD4 memory T cells, memory B cells, and macrophages comprised the major cellular fractions in the NPC microenvironment (Fig. 4A). There appeared to be significant diversity in cellular compositions between NPC tumors, including CD8 cytotoxic T cells that have been associated with improved overall survival in NPC (*32*), as well as CD4 T cells known to play a cytolytic role in viral infections and virus-driven malignancies including EBV (*33*, *34*).

Following our earlier observations of elevated CXCL11 and CCL20 transcripts in the tumor epithelial compartment, we observed a positive correlation with the expression of their respective receptors, CXCR3 and CCR6, in the microenvironment (Fig. 4, B and C). Tumor CCL20 expression was significantly correlated with increased M0 and M1 macrophage fractions (*r* = 0.458, *P* = 0.00293 and *r* = 0.377, *P* = 0.0164, respectively) in the microenvironment (Fig. 4D). Furthermore, when compared to normal nasopharyngeal epithelium, increased M0 and M1 signatures were also observed in dysplastic tissue and within the NPC tumor compartment, suggesting that these macrophages infiltrate within the tumor framework (fig. S8B). The use of a second deconvolution approach, xCell, consistently demonstrated increased macrophage infiltration within the tumor compartment, as well as a correlation between CCL20 and the M1 macrophage gene signature (*r* = 0.377, *P* = 0.0152; fig. S8, A and C). Patients with macrophage-stimulating 1 receptor (MST1R) germline mutations have been observed to be at a significantly higher risk of developing early age–onset NPC, suggesting that macrophage migration and phagocytic activity could be important protective processes. Apart from macrophages, we observed that NPC tumors and the microenvironment were also enriched for activated dendritic cells, while being depleted of immature dendritic cells (fig. S8E), consistent with observations that NPC cells are able to influence dendritic cell responses (*35*). CCL20 also correlated positively with the activated dendritic cell signature (*r* = 0.438, *P* = 0.00469; fig. S8D), supporting a critical role for the CCL20-CCR6 axis in the recruitment of antigen-presenting cells including macrophages and dendritic cells. Nonetheless, CCL20 expression in NPC tumors and in the serum of patients with NPC has been shown to be an unfavorable prognostic marker (*36*–*39*), and the roles of macrophages and dendritic cells in the NPC microenvironment remain to be further studied.

### Microdissected tumor subtypes in NPC

Unsupervised clustering of tumor epithelial gene expression libraries of pretreatment primary NPC tumor biopsies using the UMAP approach suggested the presence of two tumor clusters (fig. S9A). Patients who subsequently developed recurrent disease were observed only in cluster 1, the majority cluster comprising 72% of tumors. In contrast, none of the patients in cluster 2 (28% of tumors) developed recurrence. Differential gene expression analysis and GSEA suggested that cluster 1 tumors were enriched for processes associated with DNA replication and chromatin organization, while cluster 2 tumors were enriched for immune processes including complement activation, phagocytosis, and humoral immune response (fig. S9B). While these findings require further validation, the increased immune processes and the absence of recurrent disease observed in cluster 2 are supportive of earlier observations that tumors with increased TIL signatures have a favorable prognosis (*14*).

### The NF-κB signaling pathway in NPC

The importance of the inflammatory state is supported by whole-exome sequencing studies identifying mutations in the NF-κB signaling pathway in 7 to 41% of NPC tumors (*12*, *13*). These comprised loss-of-function mutations among the negative regulators of NF-κB signaling, including CYLD lysine 63 deubiquitinase (CYLD), TNF receptor associated factor 3 (TRAF3), and NFKB inhibitor alpha (NFKBIA), suggesting that the NF-κB pathway is activated. Because these mutations belong to both the canonical and noncanonical arms of NF-κB signaling, it remains unclear which arm of the NF-κB pathway is of greater importance in NPC. This is a crucial question as the canonical and noncanonical arms of NF-κB signaling have been described to mediate distinct and opposing functions in inflammation and cellular differentiation (*40*, *41*). Several cancers have also been observed to rely either on the canonical or noncanonical arm in particular (*42*–*44*). Nonetheless, cross-regulatory mechanisms and interdependencies do exist between both arms, allowing them to function as an integrated system (*40*, *45*, *46*).

**Fig. 5.**
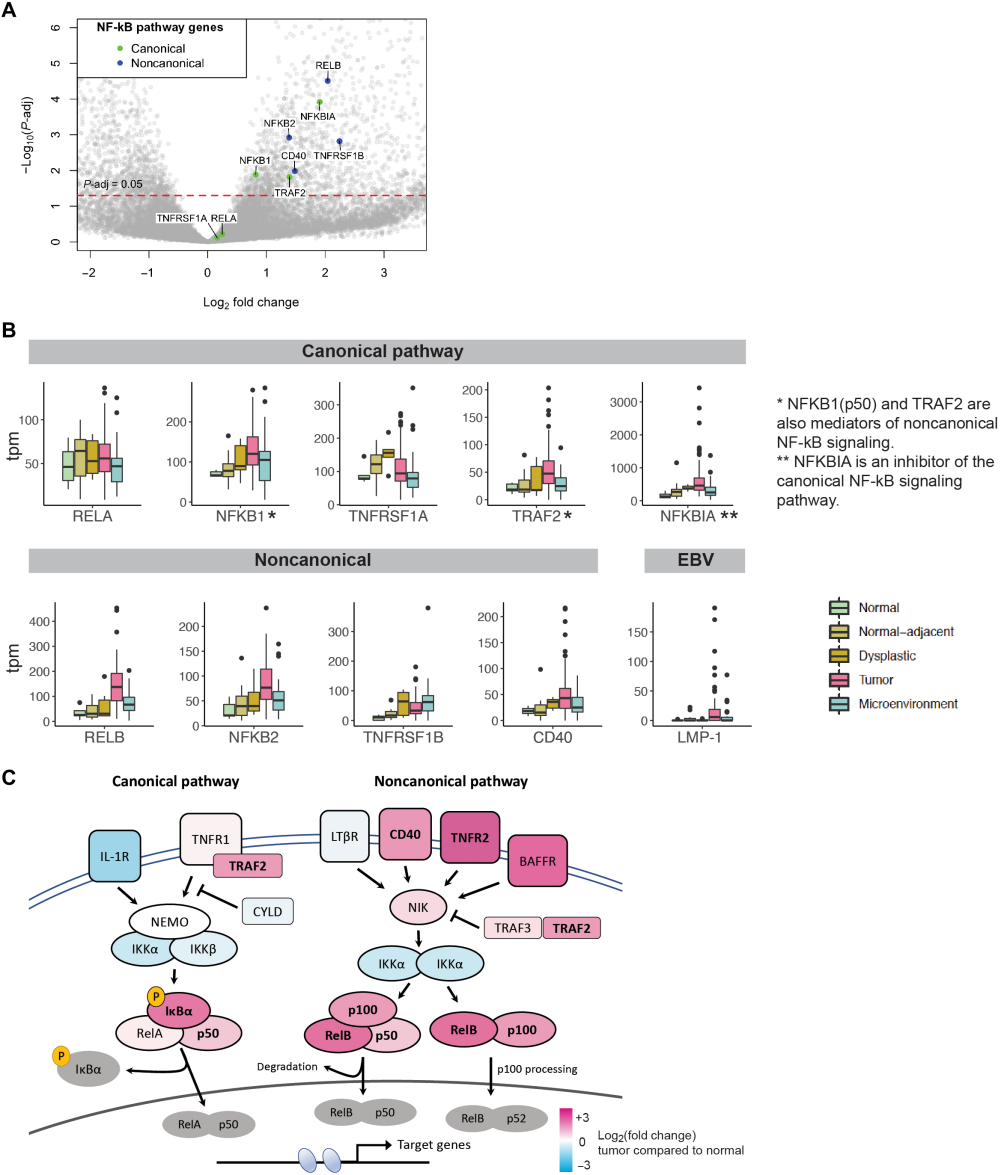
Key pathway members of noncanonical NF-κB signaling are up-regulated in NPC. (**A**) Volcano plot of differentially expressed genes comparing NPC tumors (*n* = 54 tumors) and normal nasopharyngeal epithelium (*n* = 5 controls), with key members of the NF-κB pathway highlighted. (**B**) Gene expression of key members of canonical and noncanonical NF-κB signaling (*n* = 171 libraries). (**C**) Heatmap based on fold change expression of key mediators of the NF-κB signaling pathway in NPC tumors compared to normal nasopharyngeal epithelium, with significant differentially expressed genes highlighted in bold.

Here, we observed that members of the noncanonical NF-κB pathway were preferentially up-regulated in NPC tumors compared to members of the canonical arm. These include important upstream membrane receptors CD40 and TNFRSF1B (TNFR2), as well as the critical noncanonical transcription factors RELB and NFKB2 (p100/p52) (Fig. 5, A and C). These transcripts were more highly expressed in the tumor compartment compared to the microenvironment, supporting the activation of proinflammatory changes within tumor epithelial cells (Fig. 5B). Concurrently, we observed that NFKBIA, a key inhibitor of canonical NF-κB signaling by sequestering the RelA/p50 complex within the cytoplasm, was also significantly up-regulated. Otherwise, there was no up-regulation of members of the canonical pathway apart from NFKB1 (p105/p50) and TRAF2, both of which are also recognized to mediate noncanonical NF-κB signaling (Fig. 5C).

**Fig. 6.**
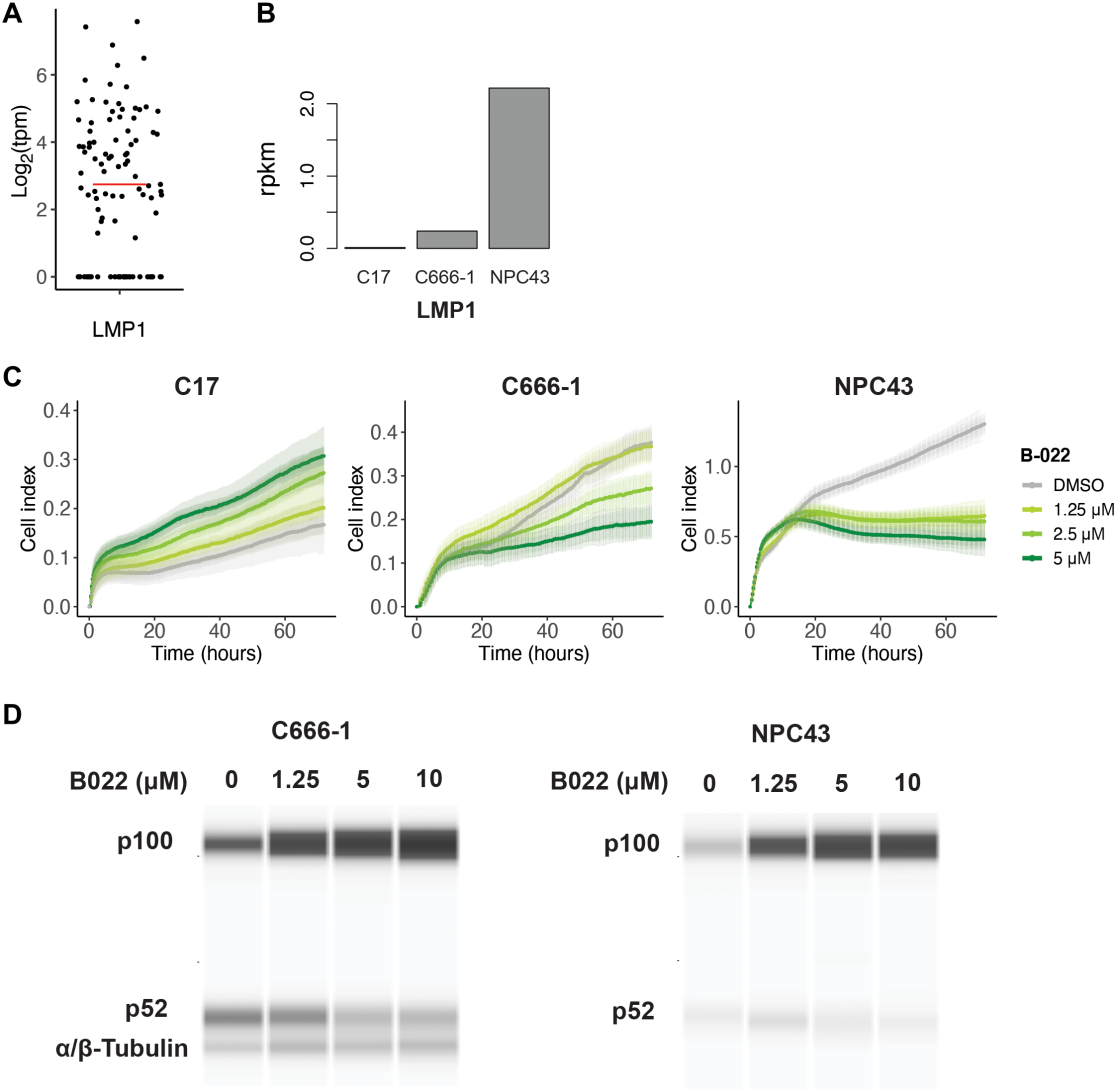
LMP1-expressing tumors are sensitive to inhibition of noncanonical NF-κB signaling. (**A**) Distribution of LMP1 gene expression in NPC tumors (*n* = 99 libraries, median log_2_tpm = 2.51, represented by red bar). (**B**) LMP1 gene expression in EBV-positive NPC cell lines (*n* = 1 per cell line). (**C**) In vitro proliferation of EBV-positive NPC cell lines when treated with B022, an NIK inhibitor targeting the noncanonical NF-κB pathway. Error bars represent SD. (**D**) Western blot of the noncanonical p100/p52 subunits in the C666-1 and NPC43 cell lines when treated with increasing concentrations of B022.

It is important to note the role of the latent membrane protein 1 (LMP1), an EBV oncogene recognized to be a regulator of both canonical and noncanonical NF-κB signaling through its CTAR2 (C-terminal activating region 2) and CTAR1 domains, respectively (*47*–*49*). Previous studies have observed the expression of LMP1 in 40 to 60% of NPC tumors based on immunohistochemistry (*12*, *50*). In this study, LMP1 transcripts were observed in 70.7% of tumor epithelial samples (70 of the 99 samples, tpm > 0; Fig. 6A). LMP1 expression correlated strongly with the expression of RELB, a key mediator of the noncanonical NF-κB pathway, as well as NFKBIA, an inhibitor of canonical NF-κB signaling (fig. S10).

We therefore hypothesized that NPC tumors might be sensitive to inhibition of the noncanonical NF-κB pathway. We tested the same panel of EBV-positive NPC cell lines (*29*, *30*) for sensitivity to noncanonical pathway inhibition using a small molecule inhibitor, B022, which inhibits NIK (NF-κB–inducing kinase)–induced p100 – p52 processing (*51*). These cell lines have varying LMP1 gene expression, with NPC43 having the highest expression, while C17 does not express LMP1 (Fig. 6B). Treatment with B022 resulted in reduced proliferation of the LMP1-expressing cell lines NPC43 and C666-1, with the highest sensitivity seen in NPC43. NPC43 and C666-1 cells treated in culture with B022 also demonstrated an accumulation of p100 and reduced p100 – p52 processing in a dose-dependent manner (Fig. 6,C and D, and fig. S11). In contrast, proliferation of the C17 cell line was not inhibited by B022 but was instead enhanced, the reason for which warrants further study. Together, these gene expression and in vitro observations suggest that the noncanonical NF-κB pathway is enriched in NPC tumors and may be of particular importance in tumors expressing LMP1.

## DISCUSSION

With the challenges of tumor purity and quantity, NPC has been a difficult tumor for genomic profiling. Earlier whole-exome sequencing studies to identify mutations in NPC have relied on stringent sample selection, microdissection, and an increased depth of sequencing to overcome such limitations (*11*–*13*). However, the sparse and varied mutational landscape of NPC posed a challenge in matching targeted therapies with potentially vulnerable tumors. Moreover, existing NPC gene expression studies are limited to microarrays (*52*, *53*) or are histologically impure (*14*, *54*) and have yielded limited impact. We therefore systematically interrogated the compartment-specific gene expression patterns in NPC with Smart-3SEQ to identify potential vulnerabilities. Our results identify new genes and pathways that are important in NPC biology and could serve as useful treatment targets.

We identify RTK ligands and mediators that are dysregulated upstream of the MAPK and PIK3CA/AKT proliferative pathways, including Sprouty proteins, FGF ligands, and galectins. The consistency in gene and protein expression among these mediators supports the hypothesis that they are working in concert to activate FGF signaling in NPC. The autocrine production of FGF and down-regulation of Sprouty proteins observed here in NPC are notably similar situations to prostate cancer, (*55*, *56*), in which clinical trials of FGFR inhibitors are ongoing. Because 66 to 70% of NPC tumors overexpress FGF1/2 based on their gene expression, it is possible that a significant proportion of NPC tumors will show sensitivity to FGFR inhibition. There are also scattered FGFR mutations and activating FGFR3-transforming acidic coiled-coil containing protein 3 (TACC3) fusions among NPC tumors, suggesting an evolutionary pressure to activate the FGF/FGFR signaling pathway (*11*, *57*); such tumors may similarly be responsive in an inhibitory strategy. We observe here in NPC cell lines that response to infigratinib is related to the expression of FGF ligands. Hence, any clinical strategy targeting the FGF pathway in NPC should take into consideration the expression of FGF within tumors as a selection criteria and the mutational status of FGFR, as early-phase clinical trials of FGFR inhibitors have found that FGFR mutations and fusions are strong predictors of clinical response (*58*, *59*).

With the importance of the NF-κB pathway suggested by earlier whole-exome sequencing studies, we shed further light on the gene expression of crucial NF-κB pathway mediators. Earlier studies exploring the susceptibility of NPC cell lines to NF-κB inhibition have focused only on the canonical arm. Here, we demonstrate that mediators of noncanonical NF-κB signaling are preferentially enriched compared with the canonical mediators. Supporting our gene expression observations, the NPC cell lines C666-1 and NPC43 showed a dose-dependent sensitivity to NIK inhibition with B022. We note that while LMP1 expression correlated with B022 response in cell lines, both C666-1 and NPC43 also harbor TRAF3 and CYLD mutations (*29*). Such a scenario is uncommon in clinical samples, where LMP1-positive tumors are mutually exclusive from tumors with NF-κB pathway mutations (*12*). Nonetheless, LMP1-positive tumors constitute a significant group of patients (40 to 60%) potentially suited for targeted therapy with NIK inhibitors currently in preclinical development (*60*–*62*). LMP1-negative tumors with TRAF3 loss-of-function mutations may also be responsive to inhibition of the noncanonical pathway.

The gene expression changes observed here across the normal to tumor spectrum, as well as the microenvironment, will be an important resource for NPC and EBV-related cancer research. Both the FGF and noncanonical NF-κB pathways identified are clinically actionable. Further work to define molecular signatures specific for each of these potential pathway subtypes, as well as the evaluation of synergistic combinations with existing therapeutic modalities, will help bring these findings forward into clinical trials for the targeted therapy of NPC tumors.

## MATERIALS AND METHODS

### Selection of cases for microdissection

Samples were collected with the approval of a Health Insurance Portability and Accountability Act (HIPAA)–compliant Stanford University Medical Center Institutional Review Board (IRB). An IRB-approved waiver of consent was obtained for genomic profiling of archival samples from the pathology tissue bank.

Panendoscopy cases comprised paraffin samples from patients who previously underwent a panendoscopy procedure to identify the primary site of metastatic squamous cell carcinoma to the cervical lymph nodes. In this procedure, biopsies are taken from multiple regions in the upper airway, including the nasopharynx. The panendoscopy paraffin samples used in this study were confirmed to be histologically normal on pathology review, and samples from any location containing malignancy were excluded.

NPC cases (*n* = 56) were obtained from paraffin blocks of NPC biopsies and two NPC tissue microarrays (TMA 308 and 340). There were 54 primary NPC tumors (including two tumors with paired recurrences) and two recurrent NPC tumors. For primary NPC tumors, only biopsy samples obtained from the nasopharynx with a diagnosis of EBV-positive NPC were included in the selection. Every case was reviewed by a board-certified pathologist to identify areas of tumor, microenvironment, dysplasia, and tumor-adjacent normal tissue.

### Microdissection of cases

Polyethylene naphthalate (PEN) membrane slides (Thermo Fisher Scientific, LCM0522) were irradiated faceup in an ultraviolet (UV) hood for 30 min. Seven-micrometer paraffin sections were mounted on the membrane slides. Slides were stored in a nitrogen desiccator cabinet until use. An adjacent 4-μm section was prepared on a glass slide and stained with hematoxylin and eosin (H&E) to be used as a reference during microdissection. Tumor and microenvironment regions were marked out by a board-certified pathologist on the H&E slide. Whenever present, dysplastic regions and normal-adjacent regions were also marked out.

Just before microdissection, membrane slides were deparaffinized with xylene and decreasing concentrations of ethanol, stained with haematoxylin (Dako, #S3309) and bluing reagent (Thermo Fisher Scientific, #7301), and then dehydrated with increasing concentrations of ethanol and xylene. Slides were dissected immediately after staining on the ArcturusXT LCM System using the UV laser to cut out each region and the infrared laser to adhere it to a CapSure HS LCM Cap (Thermo Fisher Scientific, #LCM0215). A total of 300 to 500 cells per sample was targeted.

For every NPC tumor, two samples were dissected for tumor regions, while a single sample was prepared for the tumor microenvironment, dysplastic, and normal-adjacent regions. For panendoscopy cases, a single epithelial sample was prepared for each upper airway site. In the event that the amount of cells from a single section was insufficient, additional cells from an adjacent section were included on the same LCM cap as well. All postdissection membrane slides and completed caps containing microdissected cells were reviewed on the LCM System to confirm the accuracy of the dissected regions before storing the caps in a −80°C freezer.

### Library preparation

Samples from NPC tumors were distributed over six batches for library preparation, with each batch consisting of between 29 and 38 samples. Biological duplicates of tumor regions were randomized into separate batches. Panendoscopy samples were prepared in a single batch. The C666-1 NPC cell line was used as a control and distributed across batches to confirm consistency between batches.

Libraries for sequencing were prepared according to the Smart-3SEQ protocol (*17*). This comprised the following steps: (i) lysis and 1*S* primer annealing, (ii) first-strand synthesis by reverse transcription (SMARTScribe reverse transcriptase; Clontech, #639536), (iii) 2S primer annealing and template switching synthesis of the second strand to form a double strand cDNA library, (iv) polymerase chain reaction (PCR) at 22 cycles with universal P5 primers and P7 primers labeled with unique indexes, and (v) cleanup was performed with SPRI beads (Beckman Coulter, #B23317). Specific details about the protocol may be found in the Smart-3SEQ paper.

Quality control of presequencing libraries was performed using the Agilent 4200 TapeStation with the High Sensitivity D1000 ScreenTape (Agilent Technologies, #5067-5582). Each sample intensity trace was manually reviewed, using a 165- to 1000–base pair (bp) window for evaluation. Samples with <40% of DNA within the 165- to 1000-bp window were considered unsuitable for sequencing. Quantification of libraries was performed by quantitative PCR with TaqMan Fast Advanced Master Mix (Thermo Fisher Scientific, #4444557), Illumina-specific P5 and P7 oliogoneuclotides, and the PrimeTime 5′ 6-FAM/ZEN/3′ IBFQ probe (Integrated DNA Technologies), using Kapa Biosystems DNA as standards (#KK4903). Libraries were pooled and sequenced on a NextSeq 500 machine using the High Output Flow Cell Cartridge (Illumina, #1506593).

### Computational analysis

To create a combined reference genome, the human GRCh38 genome was concatenated with the human herpesvirus 4 wild-type genome (GenBank: AJ507799.2). Annotations for the human GRCh38 genome was concatenated with annotations for the EBV genome (EBV Portal, The Wistar Institute, https://ebv.wistar.upenn.edu/tools.html).

For every read, the poly(A) tail was removed, the unique molecular identifier (UMI) was moved to the read metadata, and G-overhang was discarded using the script umi_homopolymer.py (https://github.com/jwfoley/3SEQtools). Mapping was performed by STAR Aligner (v2.7.0f) (*63*). To accommodate the shorter read lengths of Smart-3SEQ libraries, a STAR reference genome was generated using the option “--sjdbOverhang 27”. Mapping to the combined GRCh38 and EBV genome was performed using the following settings: “--outFilterMultimapNmax 1,” “--outFilterMismatchNmax 999,”, “--clip3pAdapterSeq AAAAAAAAAAAAAAAAAAAAAAAAAAAAAA,” and “--clip3pAdapterMMp 0.2.”

PCR duplicates were then marked based on position and UMI sequences using the script dedup.py (https://github.com/jwfoley/umi-dedup). Next, gene expression features were quantified using the featureCounts function from Subread (v1.6.4) (*64*).

Downstream analysis was performed in R (v3.4.4). Gene expression libraries were filtered using the following criteria: Percent uniquely mapped ≥14% and library size ≥100,000 counts. Principal components analysis did not reveal any significant batch effects or effects due to the age of the paraffin specimen (fig. S2, A and B).

Despite being distributed among different batches of library preparation and sequencing runs, gene expression libraries prepared from biological duplicates of tumor regions from the same patient appeared to be highly consistent. Tumor epithelial libraries from the same patient were found to cluster together on both principal components analysis and unsupervised hierarchical clustering (fig. S2, C and D). To perform a balanced analysis for differential gene expression between cellular phenotypes, in instances where biological duplicate libraries from the tumor compartment were obtained, only the library with the highest percentage mappability was used. A breakdown of gene expression libraries eventually included for the balanced analysis is included in table S1.

Differentially expressed gene analysis was performed using the DESeq2 package (v1.18.1) using the default Wald test and a false discovery rate of 0.05 (*65*). GSEA was performed using the package Fgsea (1.4.1), with Hallmark and GO gene sets downloaded from the Molecular Signatures Database v7.0 (Broad Institute) (*20*). Correlation analysis was performed by Pearson’s correlation using the cor.test function in base R.

The gene expression of NPC cell lines (C17, C666-1, and NPC43) was obtained starting from raw FASTQ files from published RNA-seq dataset in (*29*) using a similar mapping strategy as above. Raw reads were mapped to a combined human hg19 and EBV genome by STAR aligner. Mapping parameters were as per ENCODE RNA-seq parameters (https://github.com/ENCODE-DCC/long-rna-seq-pipeline/blob/master/DAC/STAR_RSEM.sh), with the exception of applying “—outFilterMultimapNmax 1,” to exclude any multimapping to both the human and EBV genomes. Gene expression features were quantified with featureCounts and normalized to rpkm using the EdgeR package (*66*).

### Deconvolution of immune cells in the microenvironment

Fractional deconvolution to estimate the abundance of immune cell types was performed with CIBERSORTx web interface (https://cibersortx.stanford.edu) (*31*), using the provided LM22 signature distinguishing between 22 purified immune cell types. “B-mode” batch correction was enabled to account for the different gene expression profiling techniques, while quantile normalization was disabled as recommended by the authors for the RNA-seq data. A complementary gene expression signature based–approach was performed with the xCell web interface (*24*), using the provided 64 immune and stroma cell type signature to identify enrichment of cell types.

### Immunohistochemistry

Immunohistochemistry was performed using the following antibodies in Table 1. Immunohistochemical staining was performed using VECTASTAIN Elite ABC kits (Vector Laboratories Inc.) for Mouse IgG (#PK6102) and rat IgG (#PK6104) following the manufacturer’s protocol. Briefly, following deparaffinization, rehydration, and heat-induced epitope retrieval (antigen unmasking) with 10 mM citrate pH 6 buffer (Agilent Technologies Inc., #S2369) for 3 min at 116°C. Development of stain was done using DAB (Agilent Technologies Inc., #K3468). Counterstaining was performed with hematoxylin.

**Table 1.** Antibodies used for immunohistochemistry.

**Target**	**Primary antibody**
SPRY1	Sprouty 1 antibody (H-2): sc-365520, mouse (Santa Cruz Biotechnology)
GAL3	Galectin-3 antibody (M3/38): sc-23938, rat (Santa Cruz Biotechnology)

### RNA ISH

RNA ISH was performed using the RNAscope Probe–Hs-FGF2 (ACDBio, #312111) targeting NM_002006.4, positions 1244 to 2377, and the RNAscope 2.5 HD Detection Kit (RED) for FFPE tissues (#322360) following the manufacturer’s instructions.

### Cell lines

The NPC43 and C17 cell lines were a gift from G. Tsao (Hong Kong University) (*29*, *30*). Authentication was performed at source, and the cell lines were used immediately upon receipt. All cells were cultured in RPMI media supplemented with l-glutamine (Corning, #10043CV), 10% fetal bovine serum, 1% nonessential amino acids (VWR, #116-078-721EA), and 1% penicillin-streptomycin (Thermo Fisher Scientific, #15140163). ROCK inhibitor Y-27632 (STEMCELL Technologies, #72304) was also added at a final concentration of 4 μM for the NPC43 and C17 cell lines.

### Proliferative assays

Proliferative assays were performed on the xCELLigence RTCA SP instrument (ACEA Biosciences). Cells were plated in a 96-well plate at a density of 10,000 cells per well and allowed to settle for at least 30 min before readings were obtained at 15-min intervals over the next 72 hours. Sensitivity to the NIK inhibitor, B022 (ChemFarm), was tested at 0, 1.25, 2.5, and 5 μM concentrations. Inhibitor or dimethyl sulfoxide (DMSO) was added together with basal media upon plating. All conditions were tested in quadruplicate.

### Protein quantification

To evaluate the effects of FGF2 on the Akt pathway, C666-1 cells were cultured in basal media in a six-well plate until ~80% confluent. Cells were washed twice and incubated with serum-free media containing FGF2 at 0, 20, and 80 ng/ml for 15 min. Cells were washed, trypsinized, washed, and subsequently lysed in radioimmunoprecipitation assay (RIPA) buffer containing phosphatase inhibitor (PhosSTOP, Roche) and protease inhibitor (cOmplete ULTRA, Roche). Phospho-Akt was quantified by chemiluminescence on the Simple Western WES system with the following antibodies and dilutions: phospho-Akt (Ser^473^) (D9E) rabbit monoclonal antibody (mAb) at 1:50 (Cell Signaling Technology, #4060) and HSP90 (C45G5) rabbit mAb at 1:5000 (Cell Signaling Technology, #4877).

To confirm the effects of B022 on noncanonical NF-κB signaling, C666-1 and NPC43 cells were cultured in basal media in a six-well plate until ~80% confluent. Cells were washed and incubated with basal media with B022 at 0, 1.25, 5, and 10 μM for 12 hours. Cells were washed, trypsinized (TrypLE Express, Thermo Fisher Scientific, #12-604-039), washed, and subsequently lysed in RIPA buffer (BioVision, #2114). p100/p52 was quantified by chemiluminescence on the Simple Western WES system with the following antibodies and dilutions: NF-κB2 p100/p52 rabbit antibody at 1:50 (Cell Signaling Technology, #4882) and α/β-tubulin rabbit antibody at 1:1000 (Cell Signaling Technology, #2148).

### In vivo experiments

Two million C666-1 cells were injected into the left flanks of 7- to 8-week-old NOD scid gamma (NSG) mice. At day 7, tumor establishment was determined. To ensure consistent volumes between groups, only tumors with volumes 10 to 25 mm^3^ were included. Mice were randomized into three groups and treated with infigratinib at 30 or 10 mg/kg or vehicle (10% DMSO and 90% corn oil) for 14 days by oral gavage. Serial measurements were obtained until day 21. Tumor volume (millimeter^3^) was calculated by the formula: *d*^2^ × *D*/2, where *d* and *D* are the shortest and longest diameter in millimeters, respectively.

### Data availability

Processed data from this study, including the gene expression matrix and anonymized clinical annotation, have been made available in the Supplementary Materials accompanying the manuscript. The deconvoluted immune cell scores, as well as output from differential gene analysis and gene set enrichment analysis, have been uploaded (see the Supplementary Materials) and can be accessed at Figshare (DOI: 10.6084/m9.figshare.12698687; https://figshare.com/s/bbc2e6c13cf6d4e9c336).

While the study was approved by the Stanford University School of Medicine IRB, we are unable to share the raw sequencing output files, as these samples represent old FFPE archival samples, which were not consented for sharing of genomic sequences at the time of specimen collection. We have strived to make the data widely accessible by providing the gene expression matrix (raw counts) and all other analysis output available together with this manuscript. Specific genomic processing requests can be made to the corresponding authors of this study.

### Code availability

The source code and genomic processing pipeline has been uploaded to Zenodo (DOI: 10.5281/zenodo.5347890).

## Notes

View/request a protocol for this paper from *Bio-protocol*.
