# Supplementary material for: The microdissected gene expression landscape of nasopharyngeal cancer reveals vulnerabilities in FGF and noncanonical NF-κB signaling
Source: Sci Adv. 2022 Apr 8;8(14):eabh2445. doi: 10.1126/sciadv.abh2445 (PMC8993121; doi:10.1126/sciadv.abh2445)

Science Advances
AAAS

Supplementary Materials for

The microdissected gene expression landscape of nasopharyngeal cancer reveals vulnerabilities in FGF and noncanonical NF-κB signaling

Joshua K. Tay*, Chunfang Zhu, June Ho Shin, Shirley X. Zhu, Sushama Varma, Joseph W. Foley, Sujay Vennam, Yim Ling Yip, Chuan Keng Goh, De Yun Wang, Kwok Seng Loh, Sai Wah Tsao, Quynh-Thu Le, John B. Sunwoo*, Robert B. West*

*Corresponding author. Email: rbwest@stanford.edu (R.B.W.); sunwoo@stanford.edu (J.B.S.); joshtay@nus.edu.sg (J.K.T.)

Published 8 April 2022, *Sci. Adv.* **8**, eabh2445 (2022)
DOI: 10.1126/sciadv.abh2445

The PDF file includes:

Table S1
Figs. S1 to S11

Other Supplementary Material for this manuscript includes the following:

Data S1 to S3

Table S1. Distribution of micro-dissected samples and libraries prepared. All micro-dissected libraries prepared were subject to quality control by tapestation and qPCR before sequencing, and subjected to further quality filters during bioinformatic analysis.

	NPC Tumors (n = 62)				Panendoscopy Normals (n = 5)		Cell Line Control
Cell type	Normal-adjacent	Dysplasia	Tumor	Microenvironment	Nasopharyngeal	Squamous	C666-1 in-organoid
No. of micro-dissections and libraries prepared	18	6	131	59	8	19	4
No. of libraries passing tapestation and qPCR QC	17	6	107	53	5	18	4
No. of libraries passing bioinformatics QC	15	6	99	46	5	18	4
Libraries used for balanced differential gene expression analysis	14*	6	54*	45*	5	18	NA

* after excluding biological duplicates and recurrent tumors

Figure S1. Examples of Microdissections

a. Laser-capture microdissection (LCM) of epithelia from normal panendoscopy biopsies from the upper airway **b.** Laser-capture microdissection of nasopharyngeal cancer biopsies, comprising tumor epithelial regions, tumor-adjacent microenvironment, dysplasia and tumor-adjacent normal epithelium.

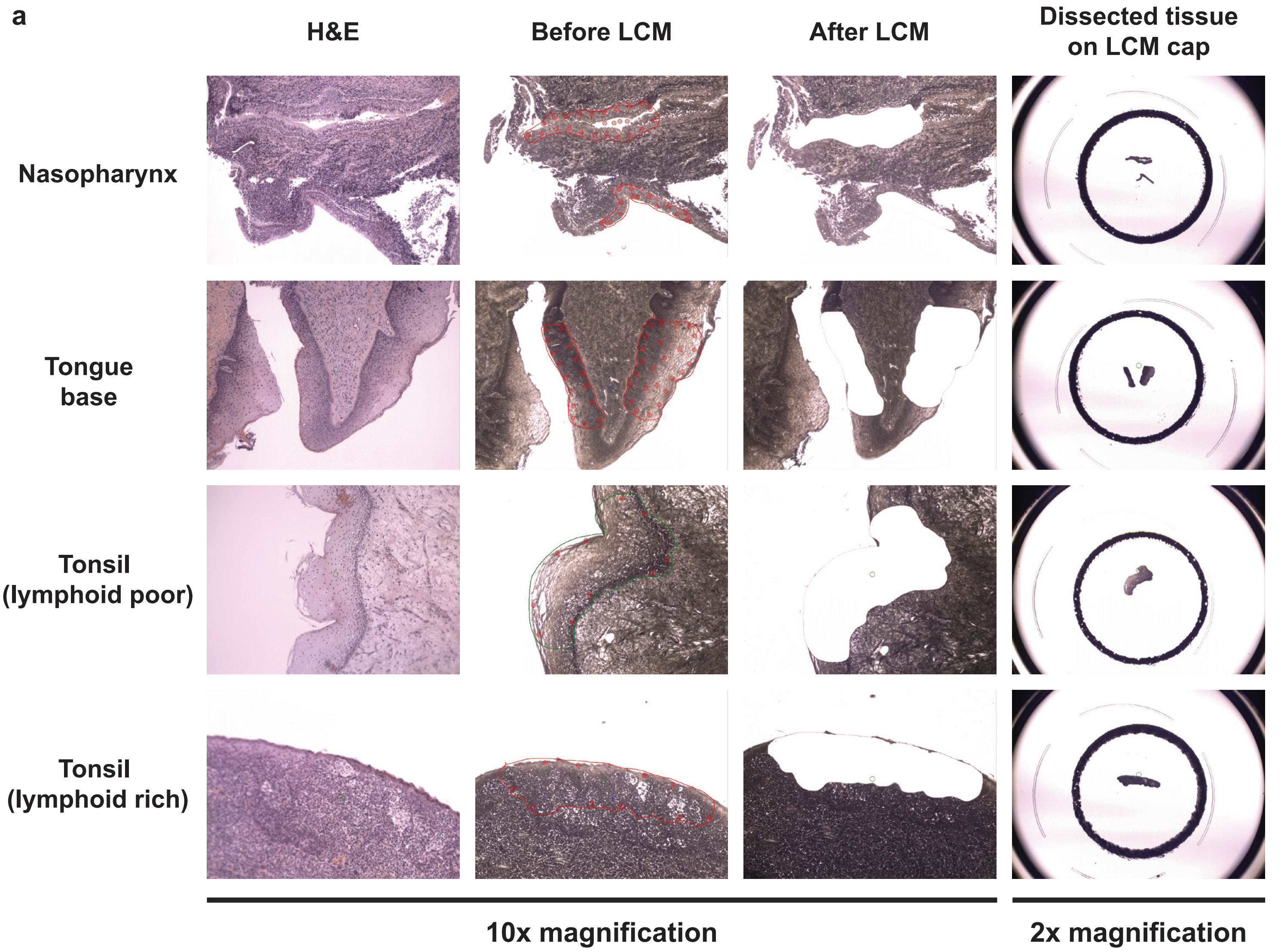

Figure S1. (continued)

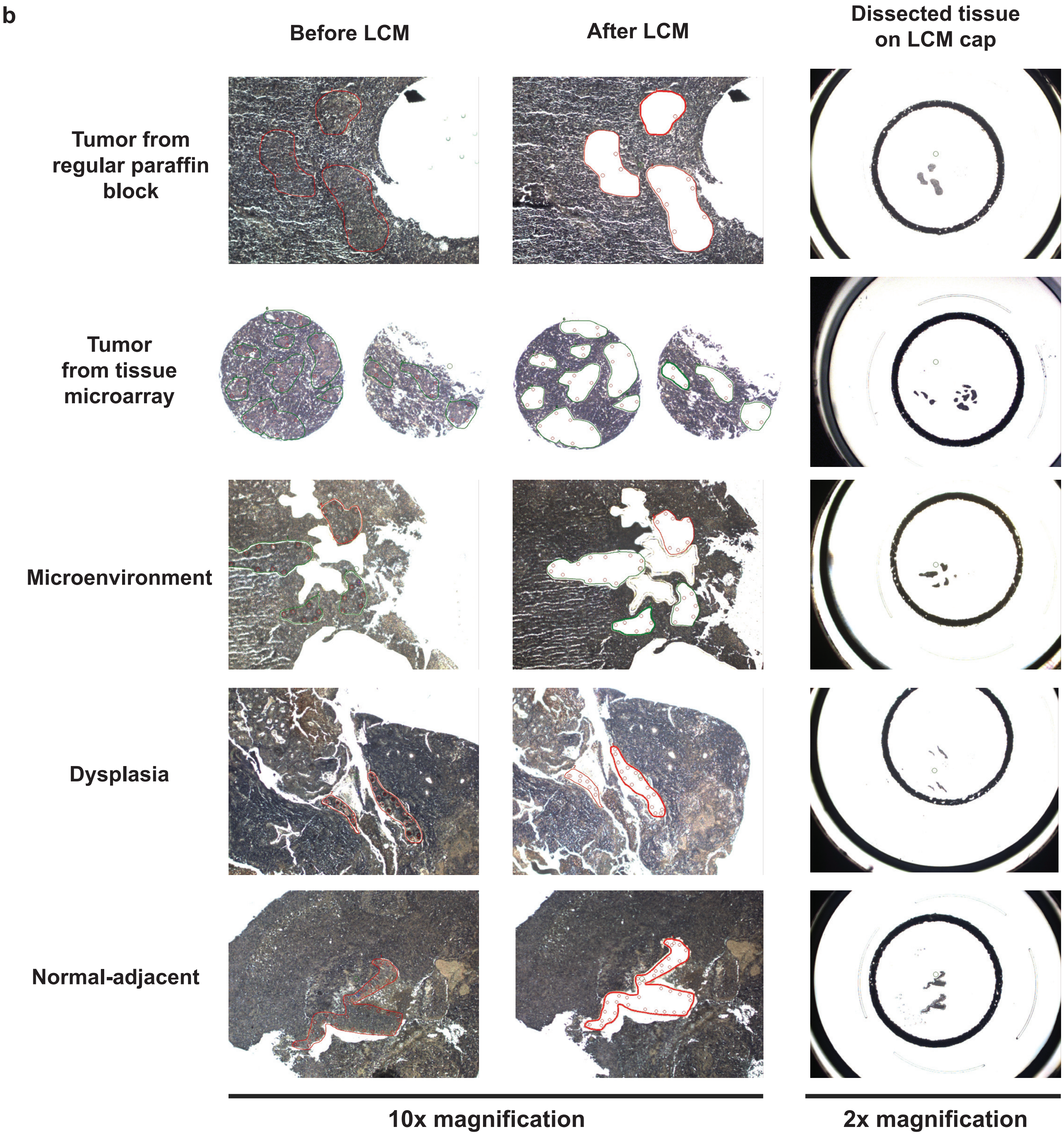

Figure S2. Principal component analysis

a. Principal component analysis of human nasopharyngeal tissue gene expression, annotated by cell-type and colored by batch of library preparation and sequencing. **b.** Principal component analysis of human nasopharyngeal tissue gene expression, annotated by cell-type and colored by age of paraffin specimen. **c**. Principal component analysis of duplicate tumor libraries (biological duplicates of the tumor comparment), labelled by source patient. **d**. Unsupervised hierachical clustering of the same libraries in (c), labelled by source patient.

a

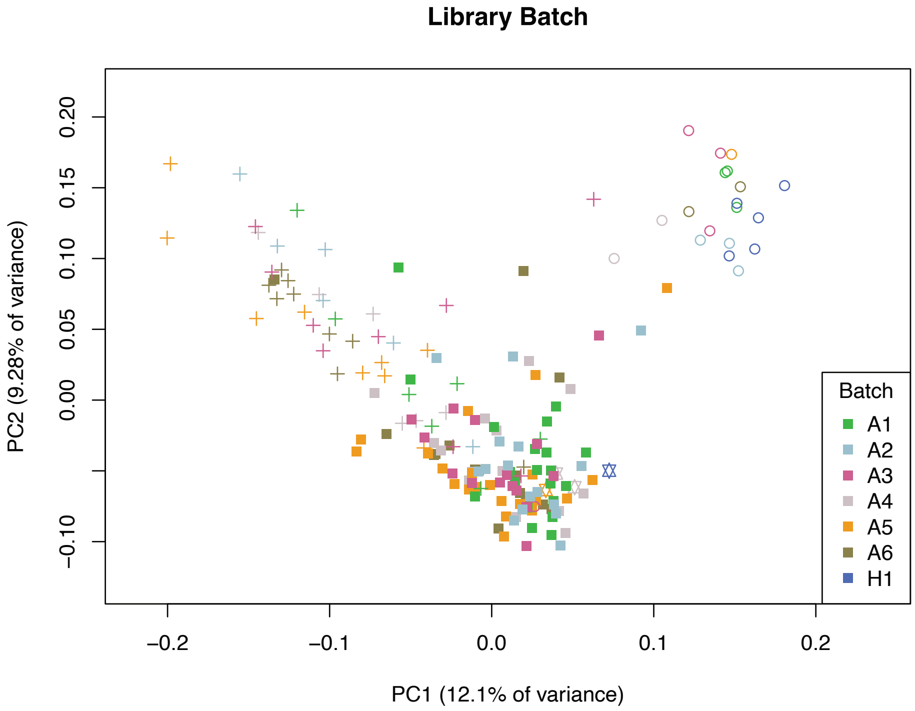

b

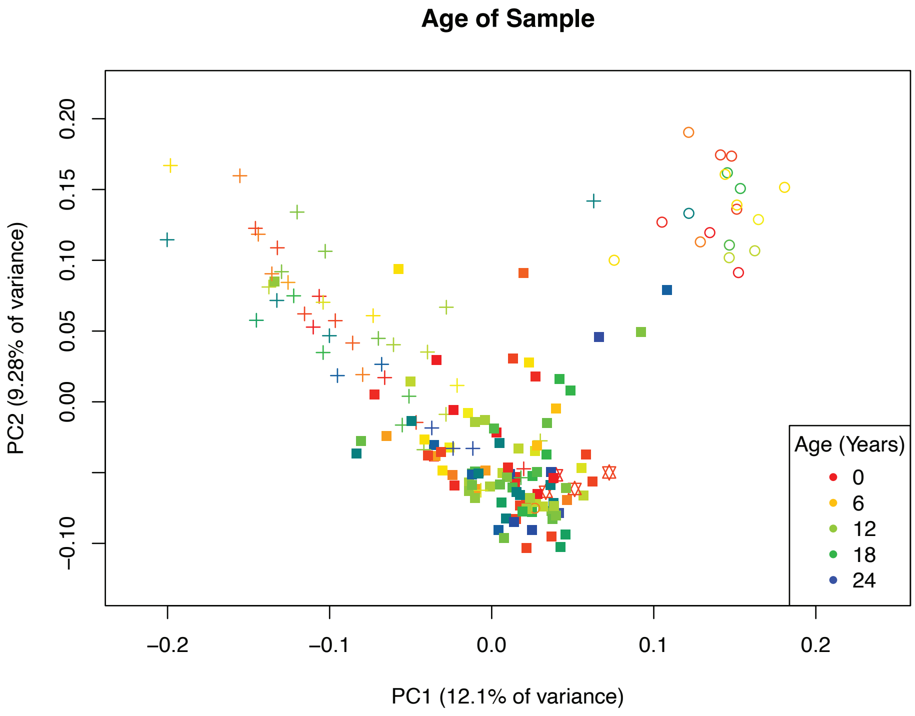

Figure S2. (continued)

c

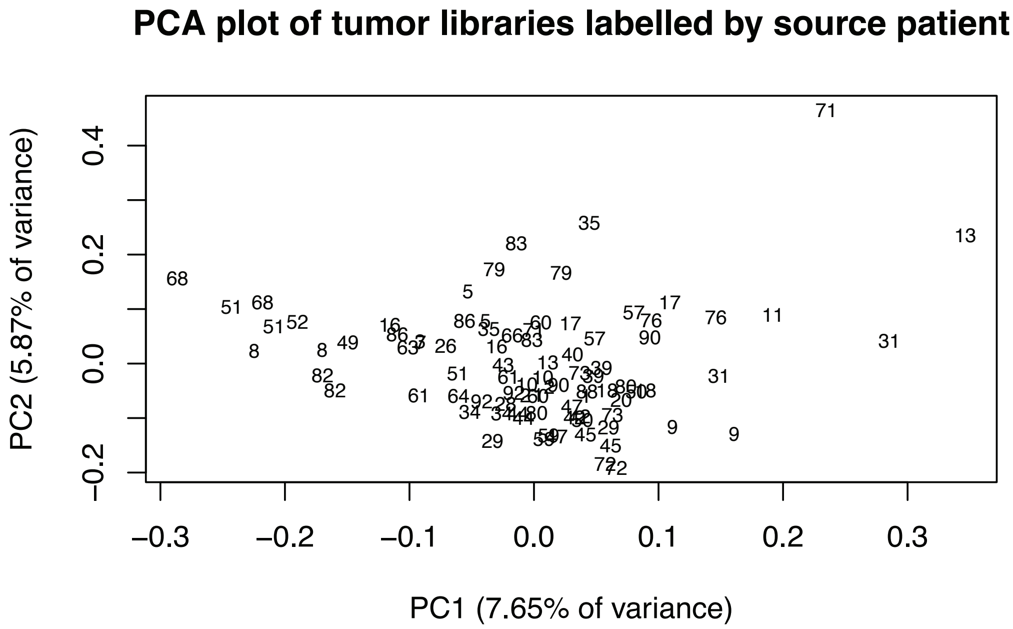

d

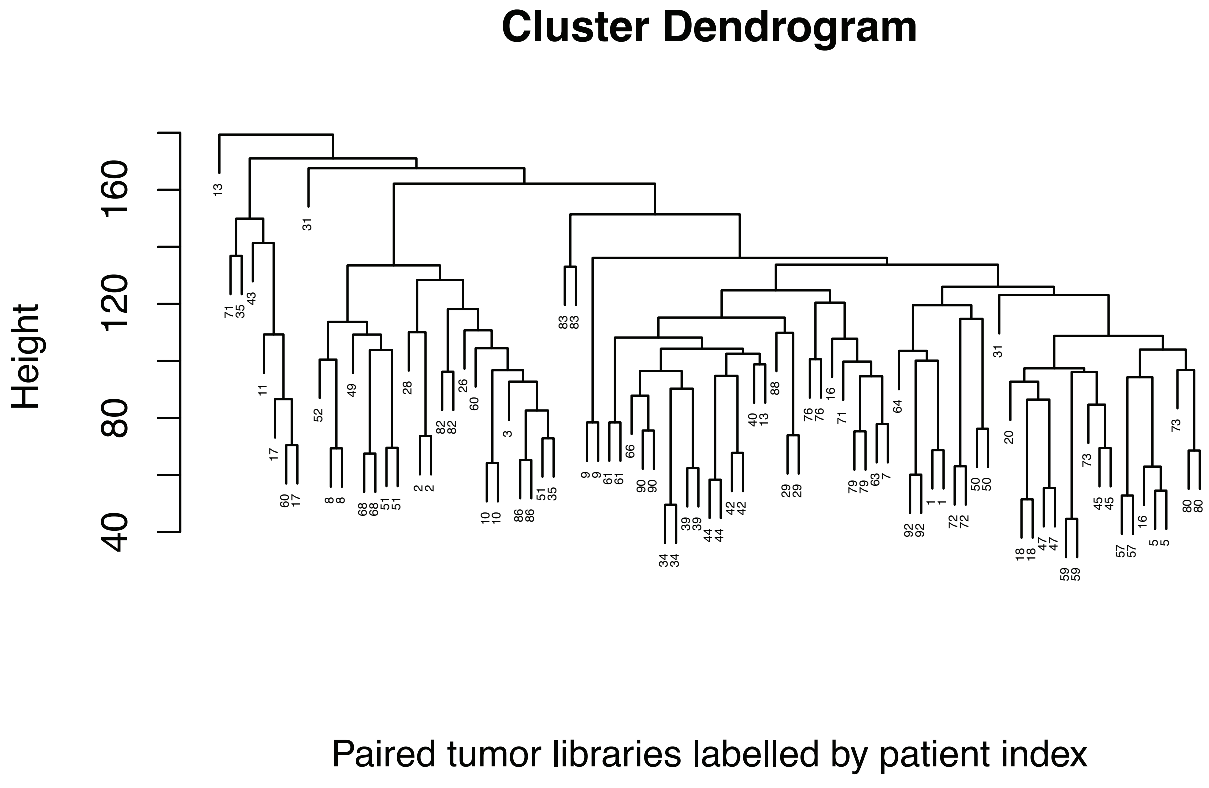

Figure S3. Hallmark biological processes

Changes in Hallmark biological processes across the transition from normal to tumor (n = 120 libraries), comparing normal-ajacent to normal epithelium (**a**), dysplastic to normal-adjacent epithelium (**b**), and tumor to dysplastic epithelium (**c**). Processes related to immune response (based on Hallmark categories) are marked in red, while processes related to proliferation are marked in blue.

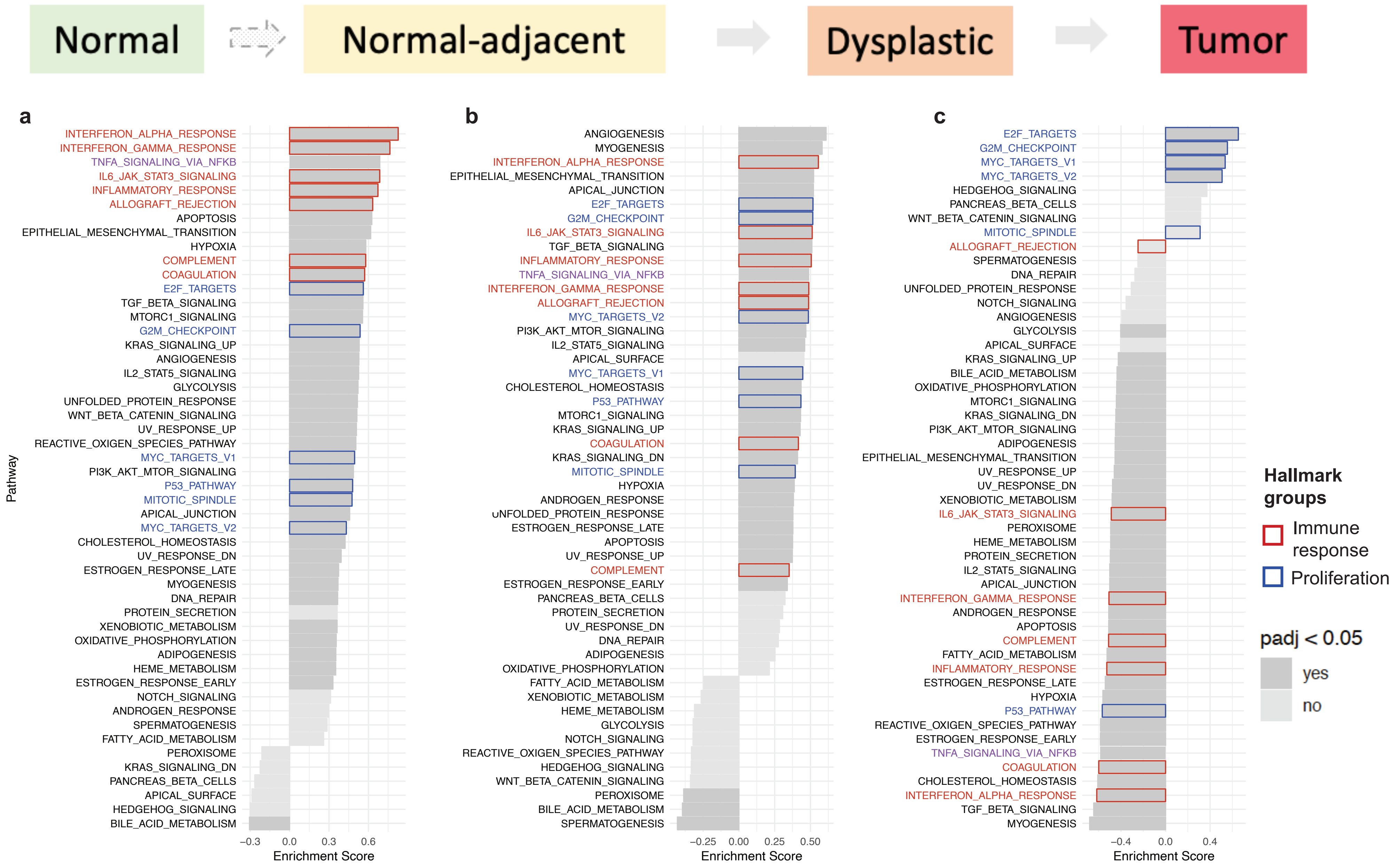

Figure S4. Differentially expressed genes in NPC tumors

a. Volcano plot of selected pathways and genes differentially expressed in NPC tumors (n = 54 tumors) compared to normal nasopharyngeal epithelium (n = 5 controls). **b**. Selected GO biological processes enriched in NPC tumors compared to normal nasopharyngeal epithelium.

a

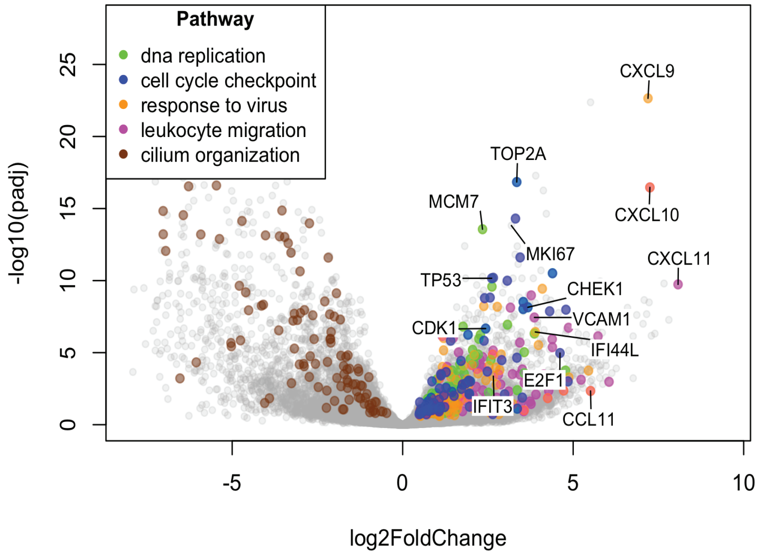

b

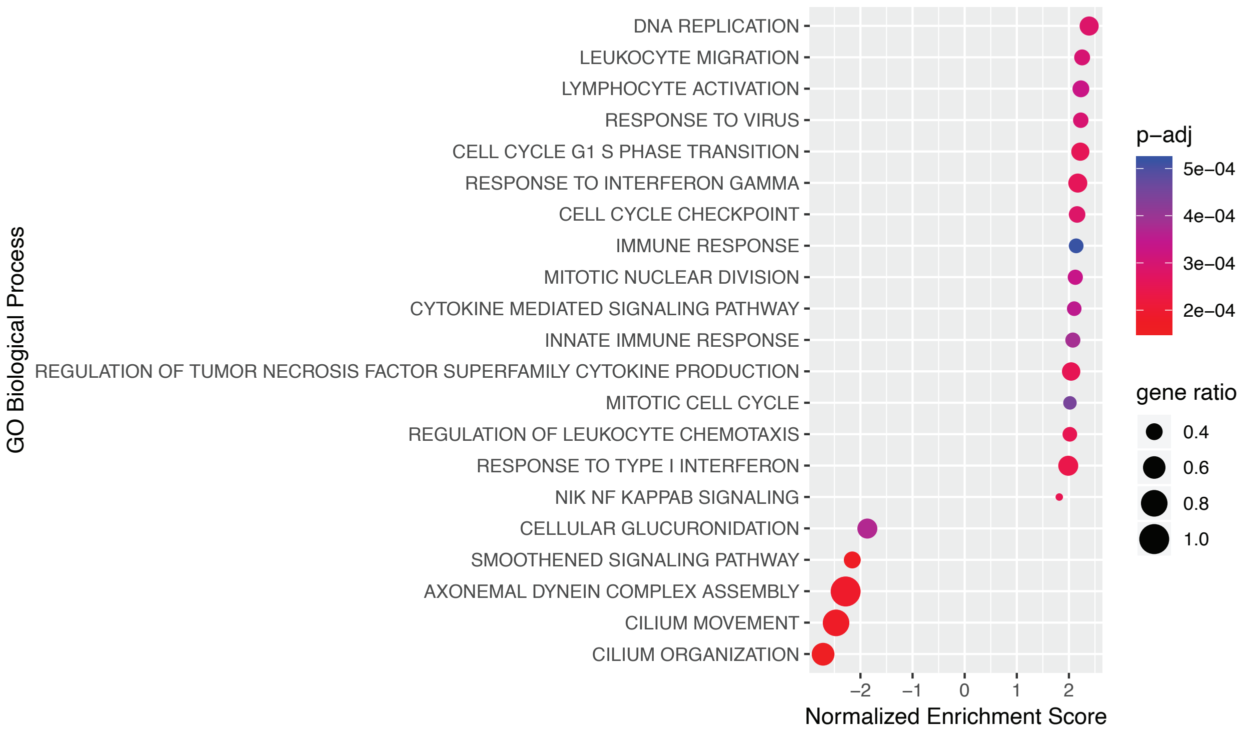

Figure S5. Differentially expressed genes in normal nasopharyngeal epithelium compared to normal squamous epithelium

a. Principal component analysis of gene expression of epithelium from multiple upper airway biopsies (n = 23). **b.** Volcano plot showing differentially expressed genes comparing normal nasopharyngeal epithelium (n = 5) with squamous epithelium from other upper airway sites (n = 15). **c.** Significantly altered GO biological processes in normal naso-pharyngeal epithelium compared to squamous epithelium. **d.** Expression of selected genes involved in lymphocyte chemotaxis in normal naso-pharyngeal epithelium (n = 5) compared to squamous epithelium (n = 15). **e.** Expression of the same chemokines across the normal – tumor spectrum (n = 171 libraries). **f.** Immature dendritic cell signature in normal nasopharyngeal epithelium (n = 5) compared to squamous epithelium (n = 15).

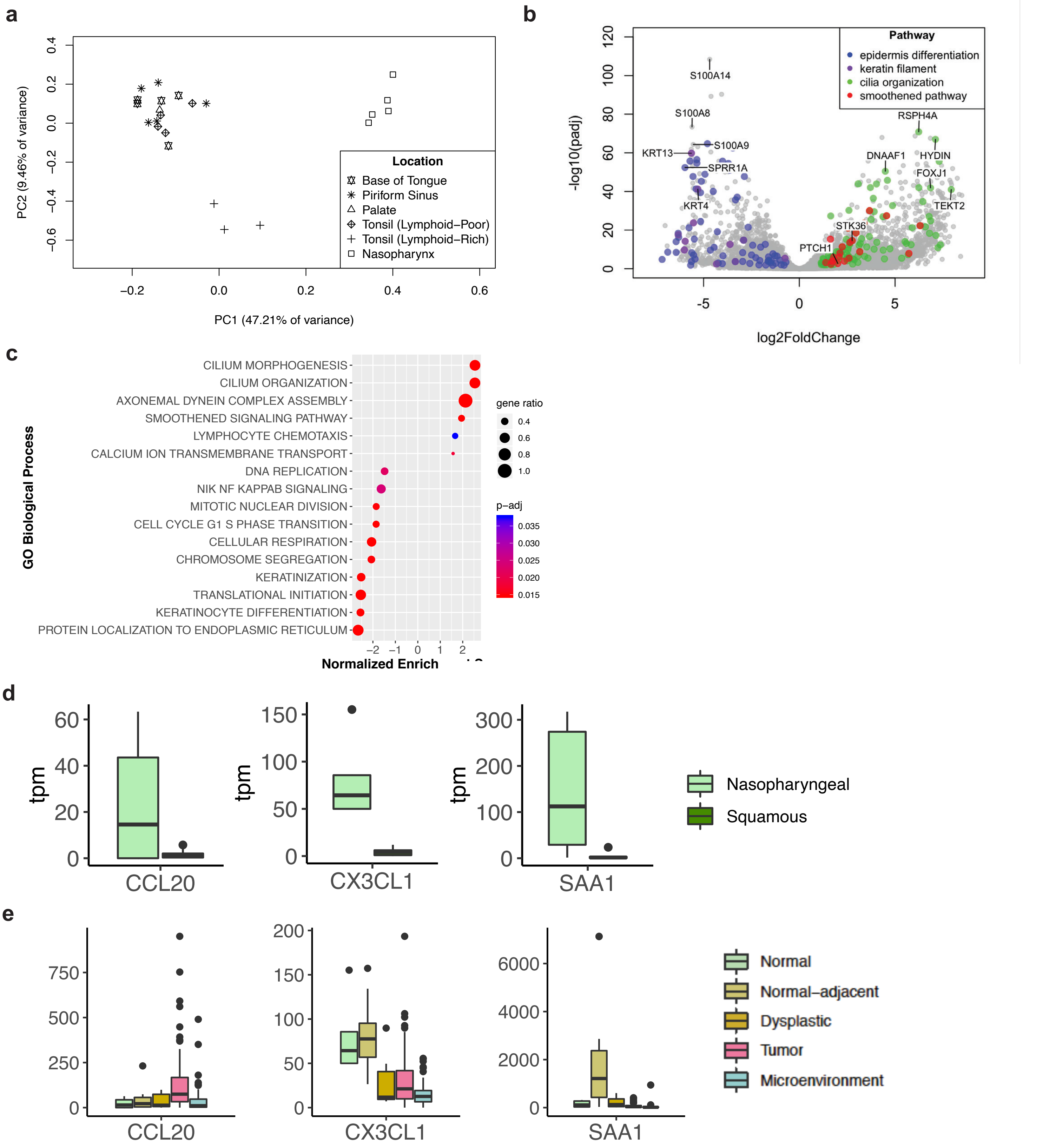

Figure S5. (continued)

f

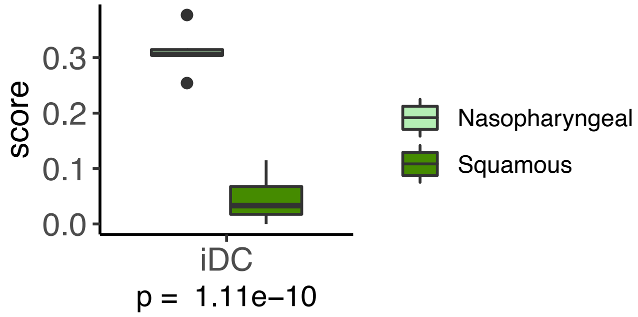

Figure S6. Expression of Galectin-1 and Galectin-3 in NPC

a. Gene expression of Galectin-1 and Galectin-3 across the normal – tumor spectrum (n = 171 libraries). **b.** Immunohistochemistry of Galectin-3 in normal nasopharyngeal epithelium and NPC tumor samples.

a

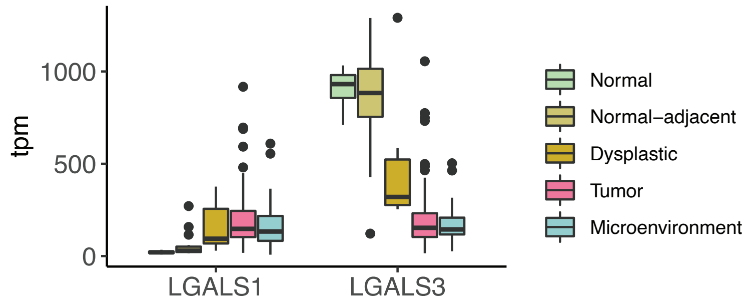

b

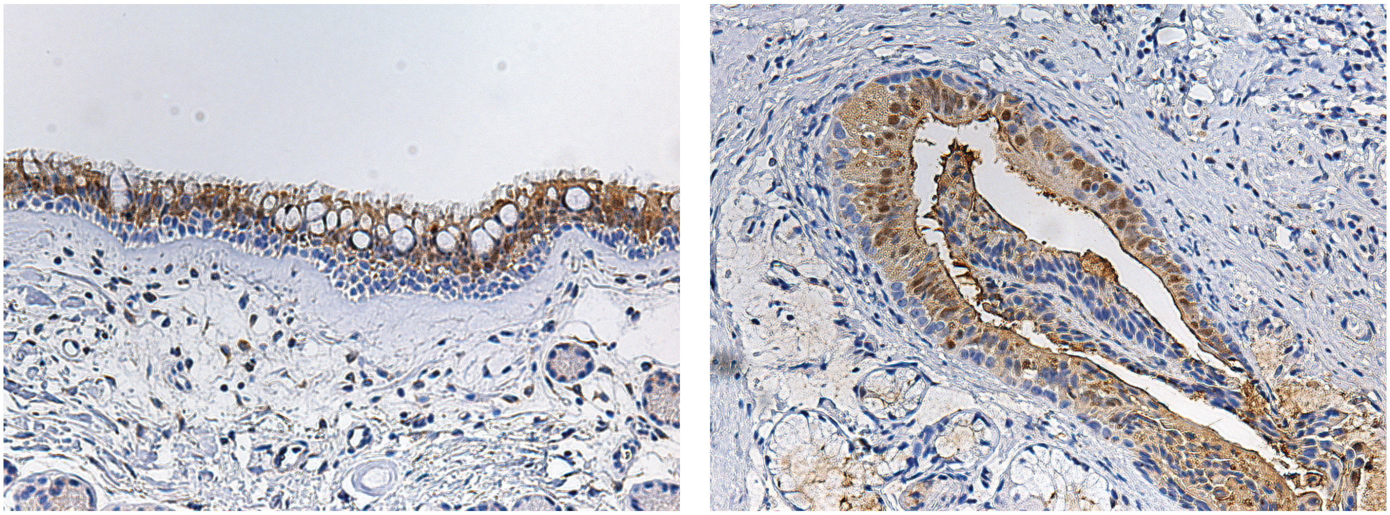

Normal nasopharyngeal epithelium from panedoscopy biopsies

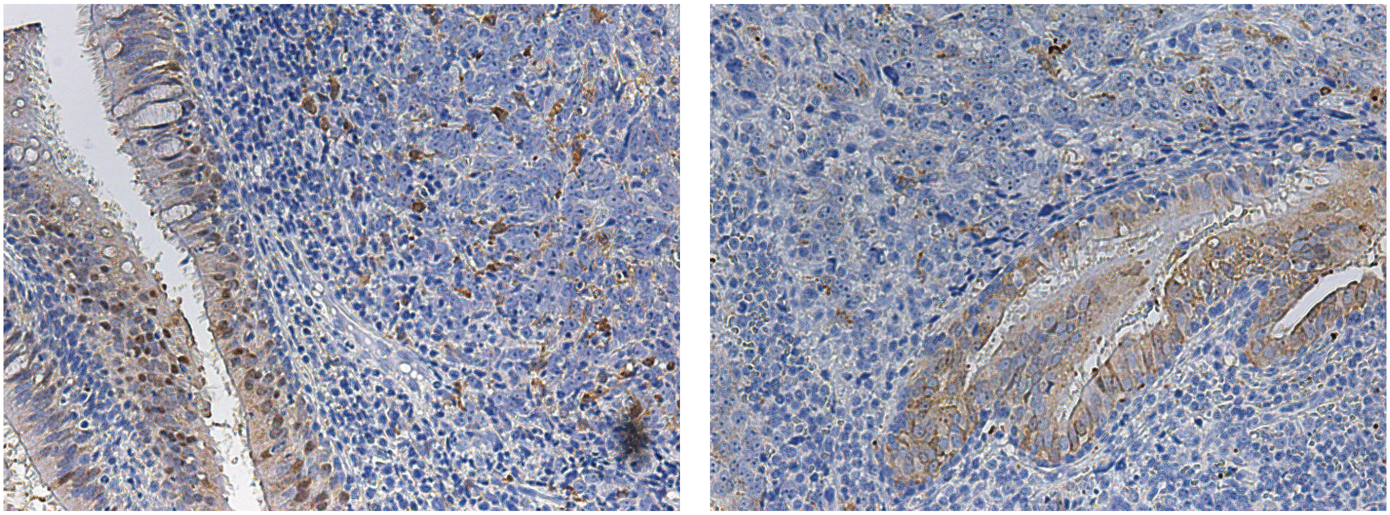

NPC tumor cells with normal-adjacent epithelium

Figure S7. Receptor tyrosine kinase ligands and receptors

a. Volcano plot comparing tumor (n = 54 tumors) and panendoscopy normal (n = 5 control) libraries, with selected receptor tyrosine kinase ligands shown. **b.** Gene expression of Sprouty-related receptor tyrosine kinase receptors and their ligands across the normal - tumor spectrum (n = 171 libraries).

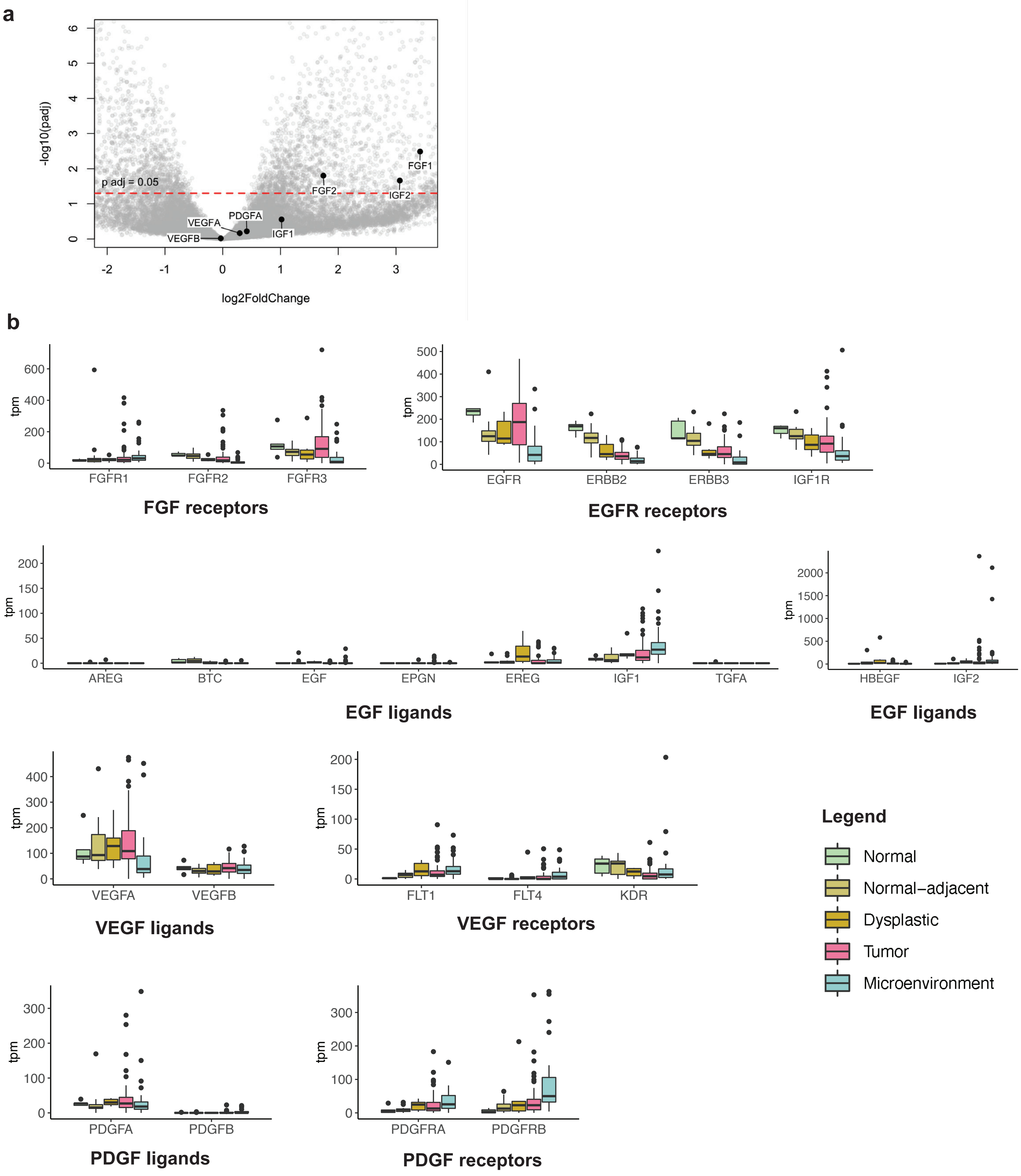

Figure S8. Tumor – microenvironment relationships in NPC

a. Correlation analysis of tumor epithelial CCL20 and xCell deconvoluted signatures for M1 and M2 macro-phages in the microenvironment (n = 41 paired tumor epithelial and microenvironment libraries). **b.** CIBERSORTx deconvoluted signatures for M0, M1 and M2 macro-phages by tissue type (n = 171 libraries). **c.** xCell deconvoluted signatures for macrophages, M1 and M2 macrophages by tissue type (n = 171 libraries). **d.** Correlation analysis of tumor epithelial CCL20 and xCell deconvoluted signatures for activated dendritic cells (aDC), conventional dendritic cells (cDC) and immature dendritic cells (iDC) in the microenvironment (n = 41 paired tumor epithelial and microenvironment libraries). **e.** xCell deconvoluted signatures for aDC, cDC and iDC by tissue type (n = 171 libraries).

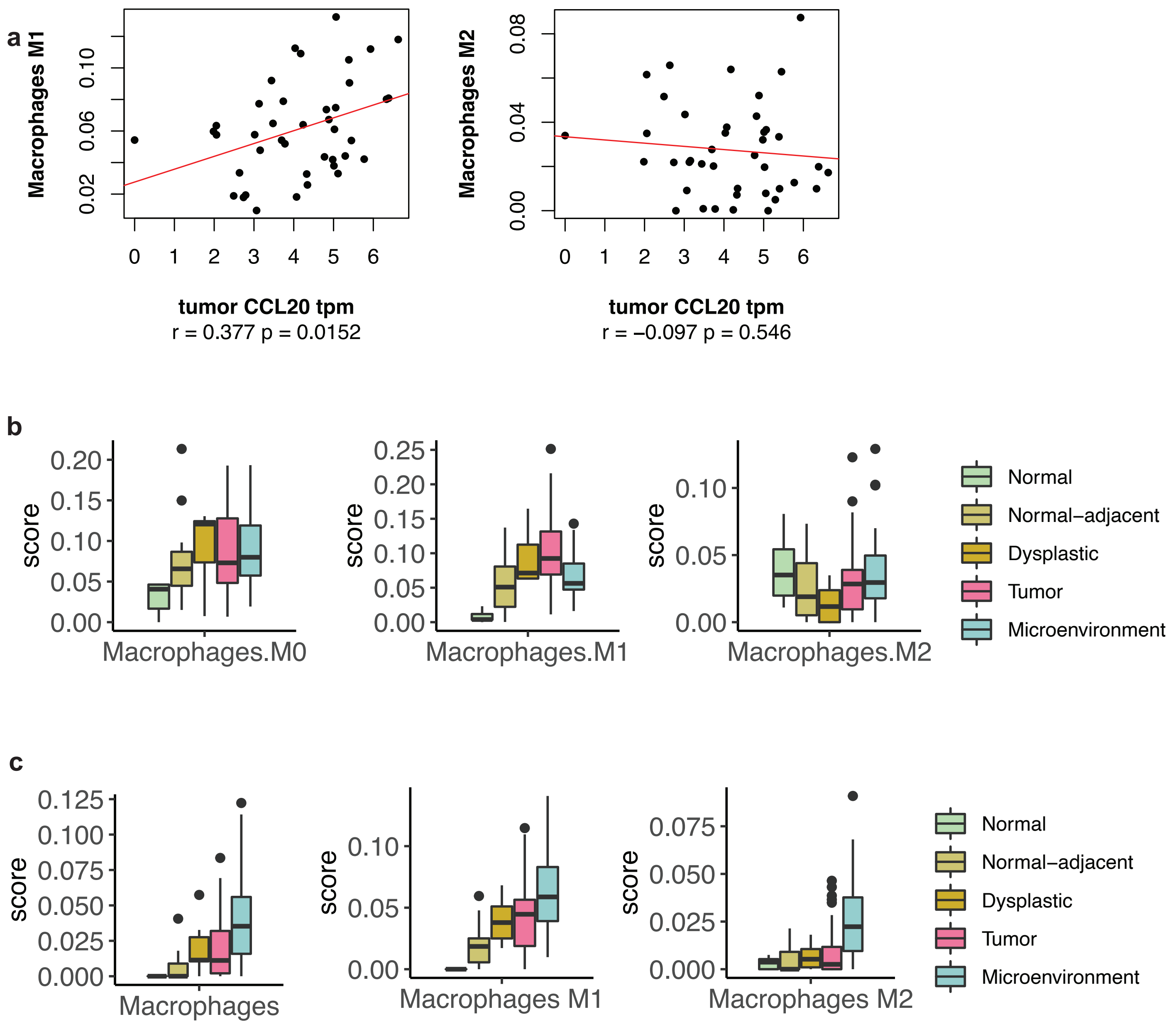

Figure S8. (continued)

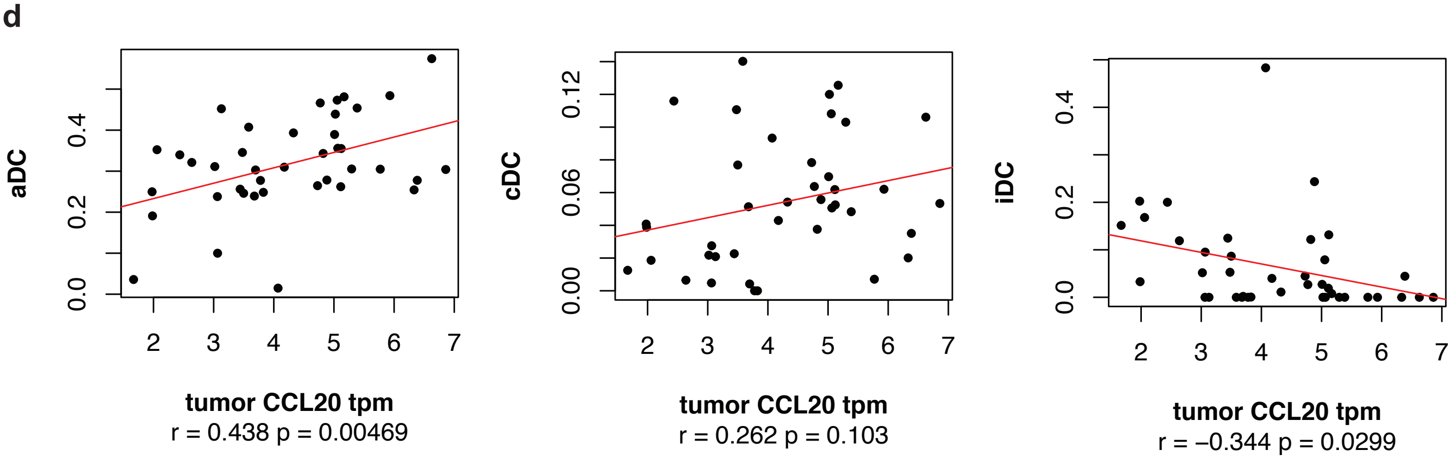

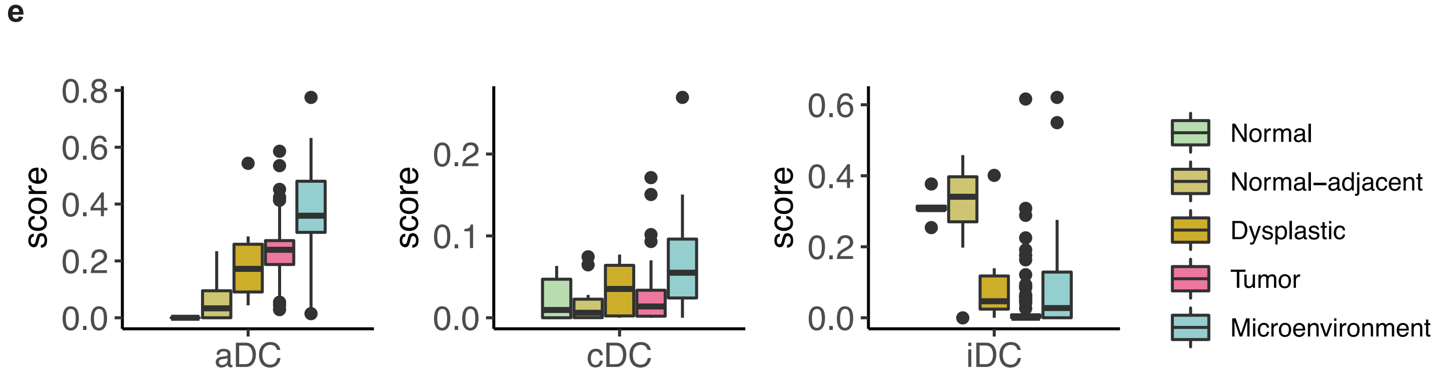

Figure S9. Tumor epithelial clusters in NPC

a. Unsupervised clustering of tumor epithelial gene expression libraries by UMAP (tumor epithelial libraries from 54 unique primary NPC tumors). Cases that subsequently recurred are highlighted in red, ellipses represent the 95% confidence limits. **b.** Volcano plot of differentially expressed genes and pathways between Cluster 1 tumors (n = 39) and Cluster 2 tumors (n = 15). **c.** Selected GO biological processes enriched in Cluster 1 tumors compared to Cluster 2.

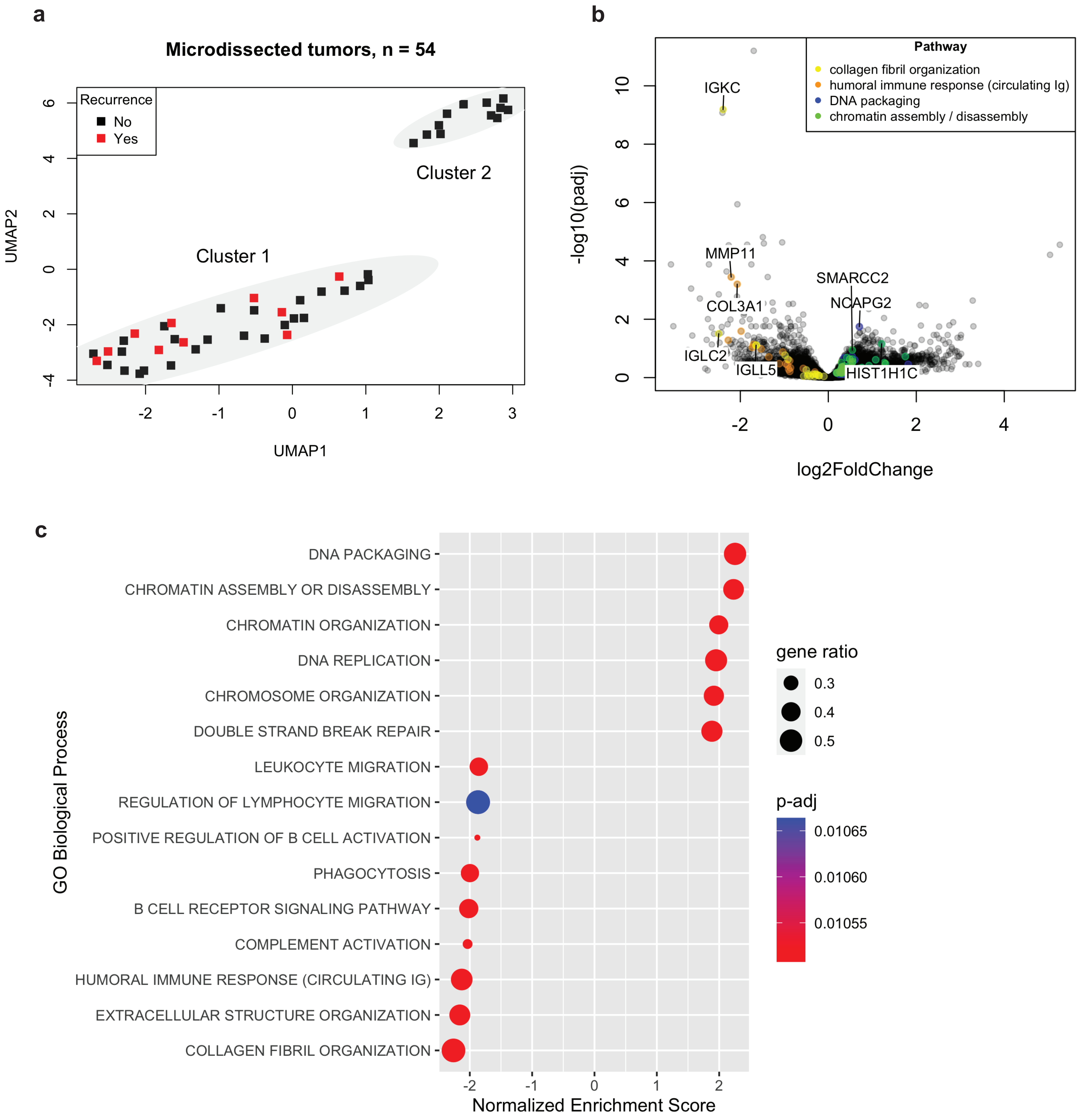

Figure S10. Correlation plots of LMP1 with key mediators of NF-kB signaling
Correlation of tumor epithelial LMP1 expression with the expression NF-kB mediators within the same tumor epithelial compartment (n = 99 libraries)

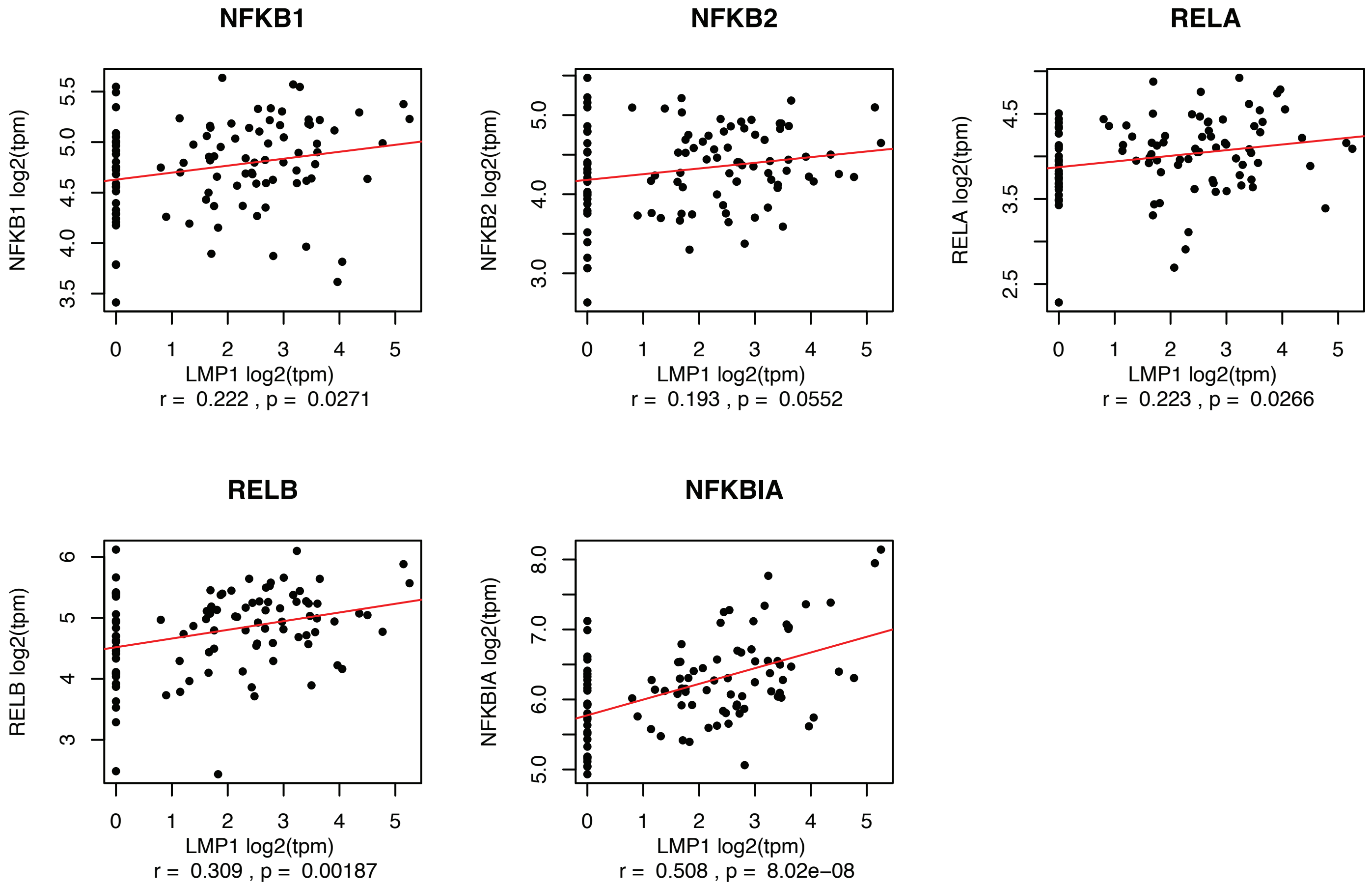

Figure S11. Chemiluminiscence plots of protein expression in NPC cell lines

a,b. pAkt and Hsp90 expression in C666-1 cells treated with FGF2 ligand. **c.** p100/p52 and α/β-Tubulin expression in C666-1 cells treated with B022, a non-canonical NF-kB pathway inhibitor. **d.** p100/p52 expression in NPC43 cells treated with B022. The left panels show the chemiluminescence intensity graph, while the right panels are a pictorial electrophoregram representation of the chemiluminescence intensity.

a

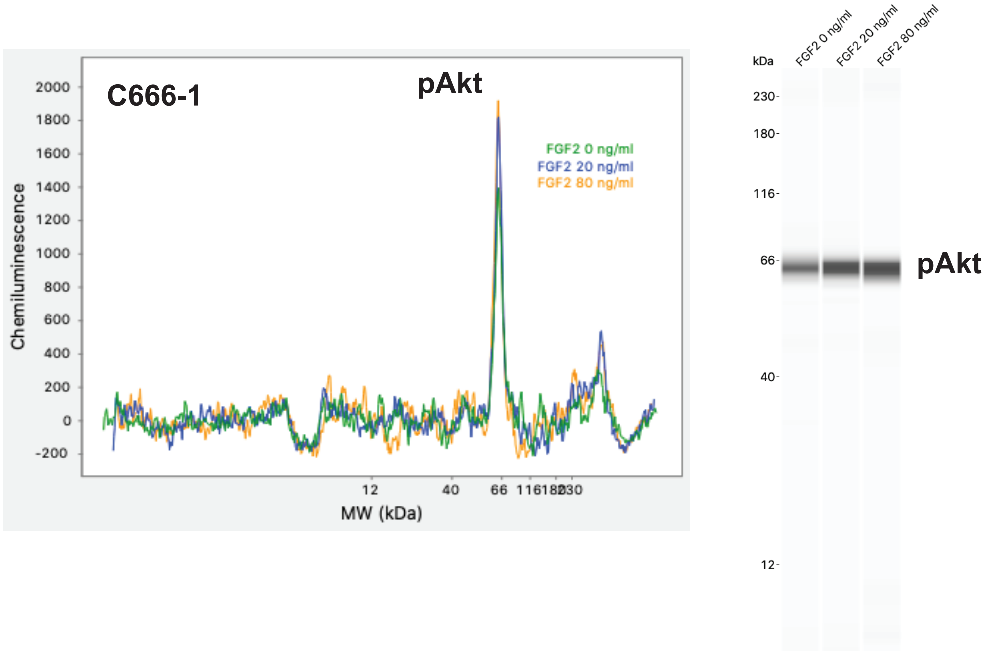

b

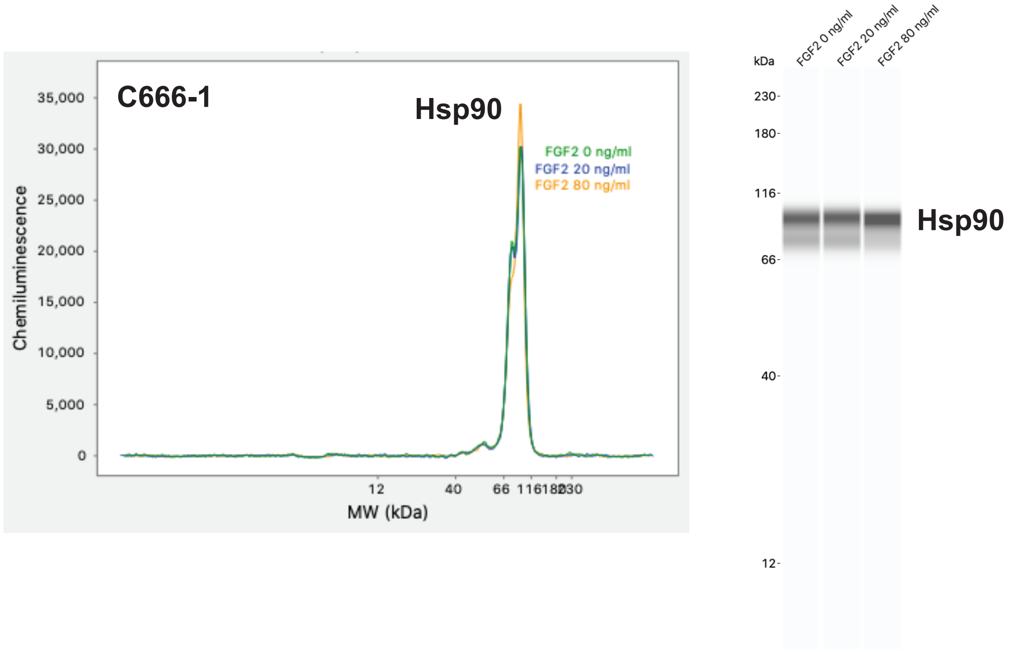

Figure S11. (continued)

c

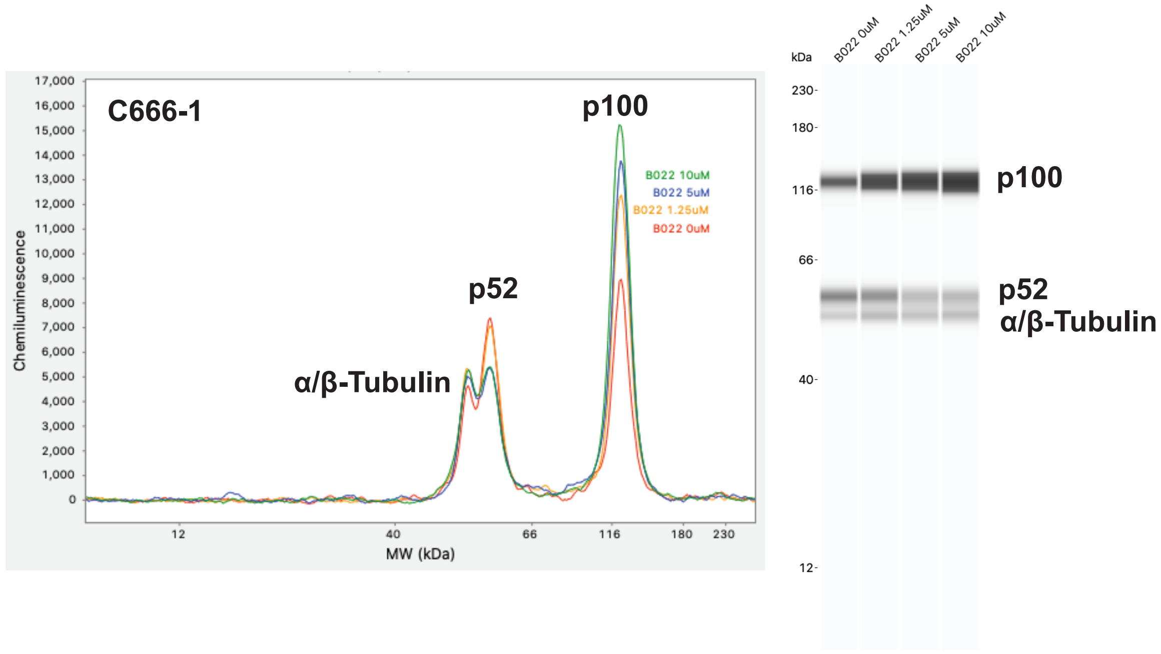

d

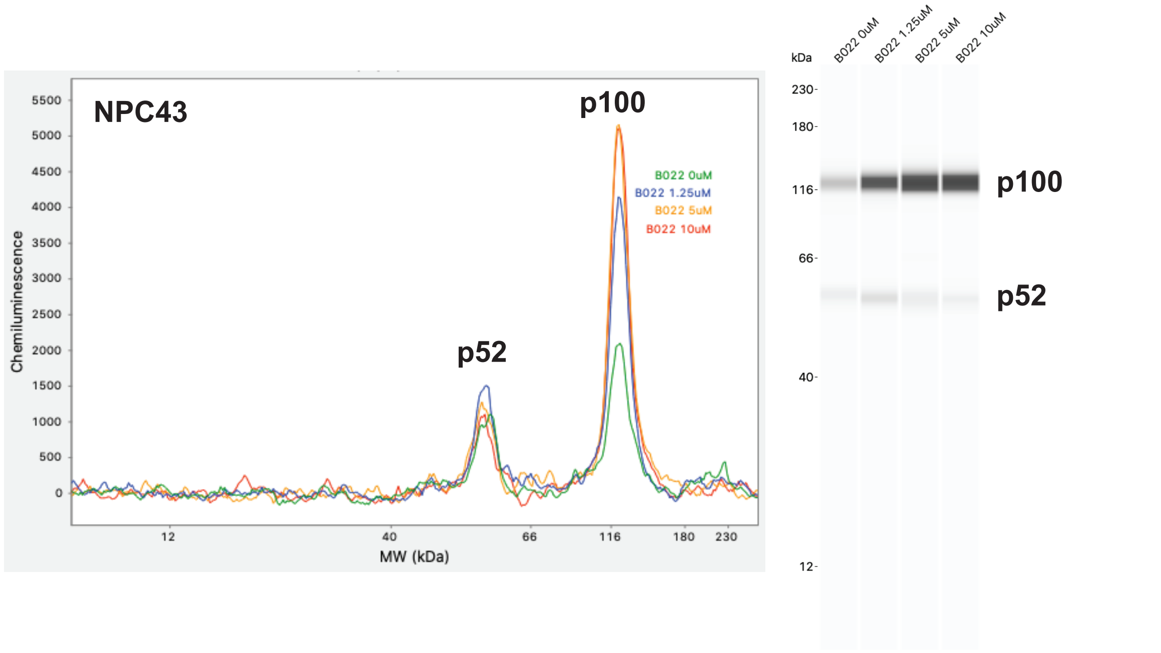

Supplement: Supplementary file 1 — Table S1 Figs. S1 to S11 [file sciadv.abh2445_sm.pdf]
